# Aging and Alzheimer’s disease: Comparison and associations from molecular to system level

**DOI:** 10.1111/acel.12802

**Published:** 2018-07-02

**Authors:** Xian Xia, Quanlong Jiang, Joseph McDermott, Jing‐Dong J. Han

**Affiliations:** ^1^ CAS Key Laboratory of Computational Biology, CAS‐MPG Partner Institute for Computational Biology, CAS Center for Excellence in Molecular Cell Science, Shanghai Institute of Nutrition and Health, Shanghai Institutes for Biological Sciences, University of Chinese Academy of Sciences Chinese Academy of Sciences Shanghai China

**Keywords:** aging, Alzheimer’s disease, cell type composition, comparison and association, epigenetics, genetic susceptibility, microglia, system inflammation

## Abstract

Alzheimer's disease is the most prevalent cause of dementia, which is defined by the combined presence of amyloid and tau, but researchers are gradually moving away from the simple assumption of linear causality proposed by the original amyloid hypothesis. Aging is the main risk factor for Alzheimer's disease that cannot be explained by amyloid hypothesis. To evaluate how aging and Alzheimer's disease are intrinsically interwoven with each other, we review and summarize evidence from molecular, cellular, and system level. In particular, we focus on study designs, treatments, or interventions in Alzheimer's disease that could also be insightful in aging and vice versa.

## 1 INTRODUCTION

Aging is the time‐dependent physiological functional decline that is also the most profound risk factor for many noninfectious diseases, including Alzheimer's disease (AD). With the growing aging population and increasing burden of health care for people with AD, research on this disease is rapidly expanding. Given the fact that Alzheimer's disease is one of the best known aging‐linked diseases, in this review, we examine how AD is linked to aging and how the research in the two fields could impact and inspire each other, at the molecular, cellular, and system level.

Alzheimer's disease was first described by the psychiatrist and neuropathologist Alois Alzheimer in 1907 as a disease that manifested extracellular amyloid plaques and intracellular neurofibrillary tangles (NFTs) in the brain, composed of abnormally folded amyloid‐β42 (Aβ42) and tau proteins, as the most pathologically important phenotypic hallmarks. Meanwhile, NFTs are universally found in all aged people and abnormally phosphorylated tau protein is already present in young individuals who do not have AD (Braak & Tredici, 2011). Amyloid plaques are also not unusual in normal brain aging. Other phenotypes of AD include neuronal dystrophy, reactive astrogliosis, synapse loss, and vascular alterations. Although much research has been devoted to biochemical mechanisms of pathogenic events induced by Aβ42 and abnormal tau, the actual cause of AD is still an open question.

## 2 MOLECULAR LINKS BETWEEN AGING AND AD

### 2.1 Genetic susceptibility

With the advent of next‐generation sequencing and genomewide association studies (GWAS), more than 20 risk loci of AD were identified (Karch & Goate, 2015). However, the identification of apolipoprotein E (*APOE*), the strongest risk factor for sporadic AD, well preceded the age of high‐throughput sequencing. Among the three common alleles (ε2, ε3, and ε4), *APOE*ε4 is associated with increased AD risk, while *APOE*ε2 is associated with decreased AD risk (Strittmatter et al., 1993). Other genetic risk loci related to AD can be classified into different functional pathways, including β‐amyloid precursor protein loading and sequential cleavage (*APP*, *PSEN1*, and *PSEN2*), cholesterol metabolism (*CLU* and *ABCA7*), immune response (*CR1*, *CD33*, *MS4A*, and *TREM2*), endocytosis (*BIN1*, *PICALM*, *CD2AP*, *EPHA1*, and *SORL1*), and others (*PLD3*). A full list of genetic risk factors identified by GWAS of AD can be found in Karch and Goate (2015).

The aging‐ or longevity‐related GWAS have generated few loci, perhaps due to the complexity of the phenotype and the lack of bona fide biomarkers of aging. However, two loci that are repeatedly found by GWAS of aging are *APOE *(Deelen et al., 2011; Ewbank, 2007; Gerdes, Jeune, Ranberg, Nybo, & Vaupel, 2000) and *FOXO3A *(Anselmi et al., 2009; Flachsbart et al., 2009; Li et al., 2009; Pawlikowska et al., 2009; Willcox et al., 2008). Similar to AD, *APOE*ε2 is found to be enriched in elderly and centenarians compared to younger individuals (Seripa et al., 2006). Elder *APOE*ε2 carriers were found to have reduced accumulation of amyloid pathology via multimodal neuroimaging (Grothe et al., 2017). Mice carrying human apoE2‐targeted replacement (apoE2‐TR) exhibited preserved memory function as compared to apoE3‐TR or apoE4‐TR mice, but this is independent with age‐related synaptic loss or neuroinflammation, and higher level of apoE and lower level of cholesterol in the cortex might contribute to this protective effect (Shinohara et al., 2016).

### 2.2 DNA methylation

DNA methylation is a well‐studied chemical modification which occurs mostly on the 5‐carbon of cytosine residues in CG dinucleotides (5mC) of DNA sequence. DNA methylation in promoter regions is correlated with transcriptional repression. Important regulators of DNA methylation include the writers (DNMT1 (maintenance methylation through DNA replication cycle), DNMT3A, DNMT3B, and DNMT3C (Barau et al., 2016) (de novo methyltransferases)), a cofactor without catalytic activity DNMT3L, and erasers (TET1–3). A distinct feature of DNA methylation in brain is that neurons are enriched in mCH (non‐CG methylation, H = A/C/T) and 5‐hydroxymethylcytosine (5hmC, established by TETs in the first of a series of stepwise modifications (Ito et al., 2011)).

DNA methylation is known to drift away from extremes of complete methylation or demethylation in older people (Heyn et al., 2012), not only intraindividual but also interindividual (Oh et al., 2016). The so‐called epigenetic clock in which age is estimated from DNA methylation data is based on the fact that epigenetic age (which is estimated from DNA methylation levels using machine learning algorithms) is highly correlated with chronological age, and thus, the deviation of epigenetic age from chronological age was found to be associated with the severity of cognitive decline in patients with AD (Levine, Lu, Bennett, & Horvath, 2015). Important to note with DNA methylation profiles in AD and brain aging is that the blood methylome might not appear to directly reflect the brain's methylome, at least from the previous data in CD4^+^ lymphocytes and dorsolateral prefrontal cortex (Yu et al., 2016). Whether this will hold for the whole brain or other brain regions needs further data for support.

Two reports (De Jager et al., 2014; Lunnon et al., 2014) established that changes in DNA methylation are associated with AD, and more specifically, a differentially methylated region in *ankyrin 1* (*ANK1*) was found to be associated with the neuropathology. A recent report (Zhao et al., 2017) also links 5hmC with AD. Epigenetic changes in AD appear to be independent of genetic variants and therefore influence disease risk by interaction with various genetic factors (Klein & De Jager, 2016). Whether age‐related changes in DNA methylation are associated with AD pathology is still unclear. An early study showed that overexpression of Dnmt3a2 isoform could improve the cognitive abilities of aged mice (Oliveira, Hemstedt, & Bading, 2012), which points to the importance of DNA methylation in cognitive function.

### 2.3 Histone modifications

Histones are the basic building blocks of eukaryotic chromatin, a complex DNA packaging system that helps organize the genome in three‐dimensional nuclear space. Histone marks are chemical modifications added to histones, including but not restricted to methylation, acetylation, phosphorylation, ubiquitination, (ADP)‐ribosylation, crotonylation, hydroxylation, proline isomerization, and sumoylation (Lardenoije et al., 2015), to control the expression of genes and to remodel chromosomes.

Chromatin changes during cellular aging are marked by loss of heterochromatin and histone (H1 tail (Funayama, Saito, Tanobe, & Ishikawa, 2006), H3, and H4 (Feser et al., 2010; O'Sullivan, Kubicek, Schreiber, & Karlseder, 2010)) in human cell lines. The overall pattern of histone modifications in aging shows a loss of repressive marks and gain of activating marks (Sen, Shah, Nativio, & Berger, 2016). Although it is not known whether such a pattern exists for in human brain aging, thanks to the Roadmap project, a snapshot of the older brain's epigenome in seven brain regions (angular gyrus, anterior caudate, cingulate gyrus, dorsolateral prefrontal cortex, inferior temporal cortex, midhippocampus, and substantia nigra) and six marks (H3K9me3, H3K27me3, H3K4me3, H3K36me3, H3K27ac, and H3K4me1) is available. We also refer the readers to reviews on other tissues or model organisms (Booth & Brunet, 2016; Lardenoije et al., 2015).

Histone acetylation is a major topic in the AD field, as it was found to be drastically decreased in both human (Zhang et al., 2012) and mouse models of AD (Gräff et al., 2012). Indirectly enhancing histone acetylation by chronic inhibition of histone deacetylases (HDACs) was able to reverse the cognitive deficits in AD mouse model (Kilgore et al., 2010) and also in aging mouse (Benito et al., 2015). This is consistent the dysregulation of H4K12ac being implicated to mediate cognitive impairment in aged mice (Peleg et al., 2010). Other histone markers are reviewed summarized elsewhere (Lardenoije et al., 2015).

### 2.4 RNA

miRNAs are a class of 22 nt length RNA molecules that negatively regulate mRNA via base pairing mostly in the 3' untranslated region. The lin‐4 miRNA was the first miRNA identified to regulate lifespan in *C. elegans*, followed by many others (Kato & Slack, 2013). The essentiality of miRNAs in neuronal cells was established by conditional *Dicer* KO, which is an important enzyme in miRNA genesis. Conditional perturbation experiments of *Dicer* in mouse brain showed progressive neuronal loss associated with behavioral deficits (Junn & Mouradian, 2012). A pool of miRNAs has been found to be associated with AD at each step of AD pathology or could serve as circulating biomarkers or in diagnostics, such as downregulated miR‐132, 212 and upregulated miR‐34a and 125b(Millan, 2017; Tan, Yu, Hu, & Tan, 2013), etc.

Long intergenetic RNAs (lncRNAs) are a >200 nt long, poorly conserved, recently discovered, abundant class of noncoding RNAs, transcribed from the intergenic and intronic regions of mammalian genome (Mattick & Rinn, 2015). A recent review summarized the lncRNAs related to senescence and aging process (Kour & Rath, 2016). However, as functions of most of lncRNAs are still unknown, the lncRNAs’ relationship to aging is limited to their expression changes during aging. In contrast to the long list of aging‐associated lncRNAs (30 lncRNAs in (Kour & Rath, 2016)), there are only six lncRNAs, BACE1‐AS, 51A, 17A, NDM29, BC200, and NAT‐Rad18, known to be associated with AD (Luo & Chen, 2016).

RNA editing is the conversion of adenosine to inosine in double‐stranded RNA by adenosine deaminases acting on RNA (ADARs) (Nishikura, 2016). In *C. elegans*, the RNA editomes of wild‐type and ADAR mutants were profiled. Worms lacking RNA editing were short‐lived, potentially due to alteration of the abundance of proteins, as determined by quantitative proteomics (Zhao et al., 2015). In AD, RNA editing was studied in a site‐specific manner (Gaisler‐Salomon et al., 2014; Khermesh et al., 2016). Recent research used a preselected set of target sites to quantify A‐to‐I RNA editing levels and found that the overall editing levels decreased in AD patients’ brain tissues, mainly in the hippocampus (Khermesh et al., 2016).

## 3 CELLULAR CHANGES IN AGING AND AD

To understand aging and AD, the well‐studied molecular changes need to be integrated with the complex cellular context of the brain. In the following section, the roles of neurons, astrocytes, microglia, and oligodendrocytes in aging and AD are examined. Compared with the drastic changes of different cell type compositions and intensive studies in AD, the mild changes of cell types in aging are understudied.

### 3.1 Neurons

Due to the prevailing neuron‐centric view and the clear importance of neuron loss to cognitive deficits, neurons have long been the main focus of brain aging studies. An early study used two‐year‐old mice (equivalent to the human age group of 65) with two subgroups (5 mice in each group) representing the worst and best performers in the Morris water maze and found that there is no loss of principal hippocampal and subicular neurons (Rasmussen, Schliemann, Sørensen, Zimmer, & West, 1996). A more recent study in human also showed that very old individuals have comparable number of neocortical neurons to younger individuals, while there is significant difference in the total number of neocortical oligodendrocytes (Fabricius, Jacobsen, & Pakkenberg, 2013). Overall, cell counting methods show that significant neuron loss does not occur during normal aging and changes are subtle and region‐specific (Burke & Barnes, 2006). A special case in successful aging is cognitive intact elderly with AD pathology, which are believed to have resistant mechanism for Aβ oligomers, and Aβ oligomers are absent from hippocampal postsynapses, while Zn^2+^ levels are lower in such cases (Bjorklund et al., 2012).

AD‐related synaptic loss occurs early in the disease process and strongly correlates with cognitive decline (Scheff & Price, 2006). High levels of Aβ have been shown to reduce glutamatergic synaptic transmission and cause synaptic loss, as shown by evidence from both in vivo and in vitro studies (Palop & Mucke, 2010). Aβ can control synaptic activity, depress excitatory transmission at the synaptic level, while triggering aberrant patterns of neuronal circuit activity and epileptiform discharges at the network level (Palop & Mucke, 2010). Using a tauopathy mouse model with in vivo intracellular and extracellular recordings, pathological tau was found to alter neocortical neuronal oscillatory patterns and firing patterns (Menkes‐Caspi et al., 2015).

### 3.2 Glia: astrocytes, oligodendrocytes, and microglia

The simple dichotomy of neuron and glia originates from the historical separation of gray and white matter based on the appearance. With the name originated from the Greek word for glue, glia were for many years viewed simply as the brain's packing material, holding neurons in place. While there have been some reports on each glia type in AD pathogenesis, astrocytes, which constitute approximately 30% of cells in the mammalian central nervous system, and oligodendrocytes, are hardly studied in the aging process. Brain aging includes proinflammatory phenotypes, altered signaling, and accumulation of senescent microglia (Harry, 2013; Mosher & Wyss‐Coray, 2014). Abnormalities in microglial cytoplasmic structure were observed in a case study of two nondemented subjects (one 68‐year‐old and one 38‐year‐old) were defined as microglial dystrophy which was further concluded as a sign of microglial cell senescence (Streit, Sammons, Kuhns, & Sparks, 2004). A transcriptional analysis of microglia from discrete brain regions at three different ages in mouse revealed that microglia aging could happen in a region‐specific manner (Grabert et al., 2016).

Reactive astrogliosis is one of the phenotypes of AD, actually noticed by Alois Alzheimer himself, but whether it is beneficial or harmful remains an open question. Astrocytes in Alzheimer's disease show changes in glutamatergic and GABAergic signaling and recycling, potassium buffering, and in cholinergic, purinergic, and calcium signaling (Osborn, Kamphuis, Wadman, & Hol, 2016). Additionally, a subtype of reactive astrocytes is induced by activated neuroinflammatory microglia, which lose most normal astrocytic functions, but gain a neurotoxic function, and is abundant in various human neurodegenerative diseases including Alzheimer's (Liddelow & Barres, 2017).

Oligodendrocytes and myelin are known targets in multiple sclerosis, a neurological disorder (Heppner, Ransohoff, & Becher, 2015). Complement‐activated oligodendrocytes are found in various neurodegenerative conditions including AD (Yamada, Akiyama, & McGeer, 1990). Overall, oligodendrocytes, although constituting ~75% of the neuroglia cells in the neocortex, are treated as a silent majority in AD research (De Strooper & Karran, 2016).

Changes related to microglia, the resident macrophages of the CNS, have long been considered to be secondary events to neurodegeneration but are now emerging as central to AD risk (Guerreiro et al., 2013; Jonsson et al., 2013; Lambert et al., 2013; Salter & Stevens, 2017; Zhang et al., 2013). GWAS repeatedly identified SNPs on TREM2, CD33, CD1, etc., which are all expressed on microglia and myeloid cells, as AD risk factors. The actual functions of those SNPs, however, are controversial in different animal models (Heppner et al., 2015). Microglia surround amyloid plaques in human AD brains. Their role in AD pathogenesis is complex and includes engulfing or degrading amyloid plaques and promoting neurotoxicity through excessive inflammatory cytokine release (Wyss‐Coray & Rogers, 2012). However, it remains unclear whether microglia phagocytose Aβ fibrils in vivo (Prokop, Miller, & Heppner, 2013). Moreover, microglial function is impaired in a progressive, Aβ‐dependent manner, as shown by a decrease in the phagocytosis of beads in a mouse model of AD (Krabbe et al., 2013). Paradoxically, microglia impairment might be sustained by inflammatory cytokines, which suggests that AD pathology can be accelerated through this vicious circle (Heppner et al., 2015). A recent single‐cell RNA‐Seq study in an AD mouse model identified a novel microglia subtype which is AD‐associated phagocytic cell conserved in mice and human (Keren‐Shaul et al., 2017). A list of attractive immune targets has been regarded as having therapeutic values (Heppner et al., 2015).

### 3.3 Neural stem cells

The adult central nervous system contains resident neural stem cells (NSCs) able to self‐renew and to generate new neurons and other neural cell types throughout life. The capacity for neurogenesis of neural stem cells diminishes with age, even in the absence of disease, as seen during bromodeoxyuridine incorporation experiment in aging rat hippocampus (Montaron et al., 1999). Age‐related decline of cognitive function is associated with decreased numbers of neural stem cells (Fan, Wheatley, & Villeda, 2017). However, recent two researches (Boldrini et al., 2018; Sorrells et al., 2018) are contradictory to each other on whether endogenous neurogenesis exists in adult human hippocampus and new techniques need to be developed to track the newly generated neurons. Stem cell‐based therapeutics, both exogenous (transplantation) and endogenous (via factors such as growth factors that stimulate stem cells), could have important implications for both aging and AD (Limke & Rao, 2002). Most recently, Zhang et al. (2017) showed that mid‐aged mice locally implanted with healthy hypothalamic stem/progenitor cells could retard aging and obtain extended lifespan, which they linked to exosomal microRNA mechanisms.

Stem cells may actually only play a limited role in AD, such that part of the neural damage observed during the disease may be attributed to the loss of stem cells’ ability to divide. Interestingly, olfactory identification deficits are suggested to be an early diagnostic marker for AD (Devanand et al., 2000) and stem cells of the subventricular zone could follow the rostral migratory stream to become interneurons in the olfactory bulb (Limke & Rao, 2002). In APPxPS1 mouse AD models, there are only limited numbers of new neurons generated and the capacity of the new granule cells is reduced in a sex‐unbalanced manner (Richetin, Petsophonsakul, Roybon, Guiard, & Rampon, 2017). NSC transplantation slowed the disease progression in an AD mouse model (Blurton‐Jones et al., 2009), while directed expression of a transcription factor, Neurod1, in cycling hippocampal progenitors could produce population of highly connected new neurons and restore spatial memory in AD mouse model (Richetin et al., 2015).

## 4 SYSTEMS LEVEL EVENTS IN AGING AND AD

### 4.1 Systemic inflammation

The “inflammaging” theory states that aging is characterized by chronic, low‐grade systemic inflammation, and a complex balance between pro‐ and anti‐inflammatory responses. Intrinsic and extrinsic factors responsible for such systemic chronic inflammation, including alterations in chronic viral infections, sex steroids, and debris from other senescent cells, are discussed in two recent reviews (Franceschi, Garagnani, Vitale, Capri, & Salvioli, 2017; Shaw, Goldstein, & Montgomery, 2013).

Although the majority of inflammation in AD is attributed to glia dysfunction, especially in microglia and astrocytes, systemic inflammation shows a strong association with AD, as shown by an interesting experiment: Under systemic immune challenge by the viral mimic polyriboinosinic–polyribocytidilic acid, mice show sporadic AD phenotypes including Aβ plaques and altered Tau phosphorylation (Krstic et al., 2012). In human, there are some similar association studies: Obesity increases the likelihood of systemic inflammation (Almond, Edwards, Barclay, & Johnston, 2013), and white‐fat tissue has a high percentage of activated macrophages secreting proinflammatory cytokines (Bastard et al., 2006); thus, it is not surprising to find that midlife obesity is a risk factor for AD (Whitmer et al., 2008). Data from microbiome studies (see below) have also provided supporting evidence on this issue. Trials in nonsteroidal anti‐inflammatory drugs on AD have shown inconsistent results with most ending in failure; however, it is speculated that this may be due to timing and choice of specific anti‐inflammatory drugs (Heneka et al., 2015; St‐Amour, Cicchetti, & Calon, 2016).

### 4.2 Cardiovascular system

The key general vascular modifications occurring during aging (Celermajer et al., 1994; Vita et al., 1990) are endothelial dysfunction and central arterial stiffness. Endothelial dysfunction may be due to diminished bioavailability of nitric oxide (Taddei et al., 2001; Tschudi et al., 1996). Arterial stiffness results primarily from loss of elastic fibers and an increase in collagen (Fritze et al., 2012). Like AD, cardiovascular disease is another aging‐related disease that brings a great burden for the patients and healthcare systems (Paneni, Diaz Cañestro, Libby, Lüscher, & Camici, 2017).

There is debate on whether cardiovascular dysfunction can induce AD, because dementia related to cardiovascular conditions can happen without amyloid accumulation, as shown by the following studies: In elderly people (sample size, 942), only midlife dyslipidemia was found to be associated with amyloid deposition, rather than other risk factors including hypertension (Vemuri, Knopman, et al., 2017); and another study from the same group (sample size, 430) found that people with cardiovascular and metabolic conditions had significantly greater neurodegeneration, but their amyloid and tau were at similar levels (Vemuri, Lesnick, et al., 2017). Another follow‐up study (sample size, 322; median follow‐up, 23.5 years) found that late‐life vascular risk factors are not associated with amyloid standardized uptake value ratios (calculated from florbetapir positron emission tomography, in this case, to reflect the brain amyloid deposition). However, when two or more vascular risk factors occur in midlife, then the odds ratio for AD increase to 2.88 (Gottesman et al., 2017).

### 4.3 Microbiome

With the advance of next generation of sequencing technology, microbes can be readily sequenced and identified. In a study of female identical twins, frailty as measured by Rockwood Frailty Index has been found to be negatively associated with gut microbiome diversity (Jackson et al., 2016). Among centenarians, the best model of “successful” aging, their gut microbiome showed high diversity of species composition compared to younger elderly adults (Santoro et al., 2017). A recent study even found that when maintained under germ‐free conditions, mice do not display an age‐related increase in circulating proinflammatory cytokine levels (Thevaranjan et al., 2017); thus, inflammaging is controllable in an animal model.

The human gut microbiome has not been investigated for associations with AD yet. In a mouse model of AD, antibiotic treatment decreases Aβ plaque deposition (Minter et al., 2016) and germ‐free APP transgenic mice have a drastic reduction in cerebral Aβ amyloid pathology (Harach et al., 2017). Poor dental status has been linked to AD or early signs of AD (Gatz et al., 2006). An early study found that subgingival microbiome is associated with changes in cognitive function (Cockburn et al., 2012). Please refer to Pistollato et al. (2016) and Tremlett, Bauer, Appel‐Cresswell, Finlay, and Waubant (2017) as a starting point.

## 5 LIFESTYLE ASSOCIATIONS AND INTERVENTIONS FOR AGING AND AD

### 5.1 Sleep

An important feature of old age is the decline in sleep, wherein non‐rapid eye movement (NREM) slow‐wave sleep (SWS) declines are especially significant. Particularly, decline in NREM sleep quality is accelerated in patients with AD relative to age‐matched normal people (Prinz et al., 1982). In addition, increased AD symptomatology is related to a parallel sleep deterioration, which appears to be associated with cognitive decline (Liguori et al.&&, 2014). For example, high tau and Aβ protein levels measured in cerebrospinal fluid were correlated with sleep impairment (Liguori et al., 2014). Besides sleep disruption, clinical sleep disorders are strongly comorbid with mild cognitive impairment (MCI) and AD. Over 60% of persons with MCI and AD have one or more sleep disturbances (Ancoli‐Israel, Klauber, Butters, Parker, & Kripke, 1991; Guarnieri et al., 2012), with sleep apnea and insomnia being most common.

Insomnia and sleep apnea are not only more prevalent in AD, but increase the risk of developing MCI and AD (Osorio et al., 2011; Yaffe et al., 2011), suggesting bidirectional links between sleep and Aβ pathology. Furthermore, individuals with sleep apnea had a younger age of MCI or AD onset (Osorio et al., 2015). By contrast, successfully treating sleep disturbance can delay the age of MCI onset (Osorio et al., 2015) and improve cognitive function in AD (Ancoli‐Israel et al., 2008; dos Santos Moraes et al., 2006). Together, these findings indicate that high‐quality sleep can mitigate Alzheimer's disease pathology. A connection between Aβ and NREM sleep has been found in rodent models (Kang et al., 2009; Roh et al., 2012). Investigating the underlying mechanisms of sleep, a recent study discovered a sleep‐dependent role for the lymphatic system in Aβ clearance (Xie et al., 2013). During NREM sleep, glial cells shrink by as much as 60%, facilitating an obviously increased flow of cerebrospinal fluid through interstitial space. This results in enhanced clearance of Aβ and other compounds. Conversely, the waking brain state can contribute to accumulation of Aβ (Kang et al., 2009), specifically through a higher neurometabolic rate relative to NREM sleep (Buchsbaum et al., 1989). Neurons consume greater levels of oxygen and ATP during wakefulness (Braun et al., 1997; Dworak, McCarley, Kim, Kalinchuk, & Basheer, 2010), while NREM sleep is associated with reduced oxygen consumption and active replenishment of ATP levels (Braun et al., 1997; Dworak et al., 2010). Waking therefore represents a state of higher oxygen, ATP, and glucose consumption, resulting in high rates of metabolic burdens (Everson, Henchen, Szabo, & Hogg, 2014). Therefore, without sufficient NREM sleep, the neurotoxicity and oxidative effects of AD pathophysiology are higher (Everson et al., 2014; Massaad & Klann, 2011; Villafuerte et al., 2015). Furthermore, Aβ accumulation is promoted by oxidative stress (Misonou, Morishima‐Kawashima, & Ihara, 2000) and further promotes oxidative stress itself. Thus, sleep loss promoted Aβ aggregation, and Aβ aggregation in turn promotes sleep loss. But, sleep loss also magnifies the effect of Aβ aggregation on neuronal function (Tabuchi et al., 2015).

Detecting selective impairments of NREM sleep quality may represent a novel, noninvasive, relatively inexpensive, and specific biomarker of AD pathology. Frequency‐specific quantitative electroencephalographic (EEG) measures of NREM sleep, particularly those in the <1 Hz signature range, may therefore represent an early biomarker of Aβ burden. Compared with other established biomarkers, sleep EEG may significantly contribute to identifying an individual's risk for developing AD years or even decades before onset of clinical symptoms (Jack et al., 2010).

### 5.2 Exercise

Physical inactivity is an important risk factor for cognitive decline in aging and for Alzheimer's disease (Norton, Matthews, Barnes, Yaffe, & Brayne, 2014). Conversely, exercise can convey a protective effect (Ahlskog, Geda, Graff‐Radford, & Petersen, 2011; Geda et al., 2012; Ngandu et al., 2015; Wirth, Haase, Villeneuve, Vogel, & Jagust, 2014) even if initiated after midlife (Tolppanen et al., 2015). In humans, higher levels of physical activity, as measured by the International Physical Activity Questionnaire, are associated with lower plasma Aβ in APOEε4 noncarriers (Okonkwo et al., 2014; Vidoni et al., 2012).

Besides physical activity, cognitive stimulation can also convey a protective effect against cognitive decline in aging and AD (Anderson‐Hanley et al., 2012; Barnes et al., 2013). Spatial navigation training in old age can protect the hippocampus from shrinkage (Lovden et al., 2012). A combination of exercise and cognitive enrichment in mice increases protective effects against synaptotoxicity of Aβ in the hippocampus (Li et al., 2013). However, the benefits from exercise or cognitive stimulation are not consistent across studies (Sexton et al., 2016), and thus, standardized protocols and outcome measures are needed for this field (Duzel, van Praag, & Sendtner, 2016).

### 5.3 Metabolism

Aging and many aging‐associated disorders involve perturbed energy balance. Metabolism, including glucose regulation and appetite balance, is controlled by both central regulatory inputs (primarily via the hypothalamus) and peripheral signals such as insulin, ghrelin, cholecystokinin, and adipokines (e.g., leptin and adiponectin). It is possible that the association between increased risk of developing AD and excess body weight reflects the potential effect of a diet high in simple sugars and fats to the development of AD. A recent study showed reducing caloric intake increases healthspan, reduces damage in the brain due to aging, and provides greater maintenance of various brain functions (Martin et al., 2008; Martin, Golden, Egan, Mattson, & Maudsley, 2007; Martin, Ji, Maudsley, & Mattson, 2010; Martin, Mattson, & Maudsley, 2006).

There is also a connection between type 2 diabetes mellitus (DM) and AD, and DM individuals have ~2‐fold increase in risk of developing AD, compared to patients without the condition (Ott et al., 1999). Furthermore, in the same study, DM requiring insulin treatment was associated with a fourfold increase in incidence of AD. The presence of type 2 DM and the APOEε4 allele together has also been shown to increase the risk of developing AD, to >5‐fold, compared to individuals without those two conditions (Peila, Rodriguez, & Launer, 2002). Tau phosphorylation is increased in the cortex and hippocampus of type 2 diabetes mice compared with controls. Recent work has begun to uncover the underlying mechanisms of insulin signaling dysfunction in AD. Clinically, there is a higher density of insulin receptors in the brain of patients with AD compared to control subjects, possibly reflecting upregulation of the receptor in an attempt to compensate for the decreased functionality of insulin (Frolich et al., 1998). By contrast, some studies reported decreased insulin receptor binding in individuals with AD in comparison with age‐matched control (Arnold et al., 2018; Rivera et al., 2005; Steen et al., 2005). For example, reduced insulin, insulin receptor, IGF1 and IGF2, reduced total IRS1 mRNA expression, and reduced protein indicators of downstream insulin signaling activity (including p85‐associated IRS1, phosphorylated AKT) have been reported in postmortem AD brain (Steen et al., 2005). Although there are some controversial results about insulin receptor concentration, insulin resistance in AD has been demonstrated a novel ex vivo insulin signaling stimulation experiment (Arnold et al., 2018; Talbot et al., 2012). Insulin may affect Aβ degradation via an insulin‐degrading metalloprotease. It has been observed that the decreased activity, low concentrations, and small amounts of mRNA of insulin‐degrading enzyme in brains of patients with AD and knockout mice that lack the enzyme have reduced degradation of Aβ and insulin in brain (Lam & Lu, 2007; Li et al., 2002; Shanley, Irving, & Harvey, 2001). Similarly, insulin resistance is commonly observed in older adults (Barzilai, Huffman, Muzumdar, & Bartke, 2012; Morley, 2008). Insulin resistance usually caused the unrestrained hepatic gluconeogenesis, adipose lipogenesis, and defective glycogen synthesis and glucose uptake in skeletal muscle. In addition, the proinflammatory cytokines, which is increased with aging, are involved in insulin action (Sepe, Tchkonia, Thomou, Zamboni, & Kirkland, 2011). Adiponectin is also a metabolic regulator that can be linked to aging due to its effect on insulin sensitivity (Berg & Scherer, 2005). Meanwhile, aging is strongly associated with a progressive loss in mitochondrial function (Dirks, Hofer, Marzetti, Pahor, & Leeuwenburgh, 2006). Besides insulin, it was found that lower plasma leptin levels were associated with a higher risk of incident AD (Lieb et al., 2009). In addition, glucagon‐like peptide 1 can reduce Aβ levels in vivo and decreases levels of amyloid precursor protein in cultured neuronal cells (Perry et al., 2003).

Brains with AD display a higher occurrence of “adipose inclusions” or “lipoid granules”, suggesting aberrant lipid metabolism (Foley, 2010). In the brain, cholesterol is present mainly in its unesterified form in myelin sheaths and the cellular membranes of glial cells and neurons (Dietschy & Turley, 2001). As the blood‐brain barrier limits efficient exchange between brain and plasma lipoproteins, the majority of brain cholesterol is derived from de novo biosynthesis, rather than from plasma LDL (Dietschy & Turley, 2001). Excess free cholesterol in the cell is converted into cholesteryl esters by the enzyme sterol O‐acyltransferase 1 (ACAT1; also known as acyl CoA:cholesterol acyltransferase 1), followed by accumulation in intracellular lipid droplets or efflux through the plasma membrane into the extracellular environment (Chang, Chang, Ohgami, & Yamauchi, 2006). Increasing levels of cholesteryl esters enhance Aβ release in cultured cells, whereas pharmacological inhibition of ACAT1 (for example, CP‐113, 818 treatment) can lead to the reduction in both Aβ and cholesteryl ester (Bhattacharyya & Kovacs, 2010; Hutter‐Paier et al., 2004; Puglielli et al., 2001).

Cholesterol regulates Aβ generation through regulating secretase activities. By reducing cellular cholesterol level through lovastatin and methyl‐β‐cyclodextrin, the formation of Aβ is inhibited (Hartmann, Kuchenbecker, & Grimm, 2007; Simons et al., 1998; Vetrivel & Thinakaran, 2010). Cholesterol levels can also directly regulate β‐secretase‐mediated production of Aβ (Fassbender et al., 2001; Simons et al., 1998; Wahrle et al., 2002). Inclusion of cholesterol or sphingolipids in phosphatidylcholine‐containing vesicles leads to increased γ‐secretase activity (Osawa et al., 2008; Osenkowski, Ye, Wang, Wolfe, & Selkoe, 2008). Cholesterol depletion also decreases the association of BACE1 with lipid rafts resulting in decreased processing of APP (Ehehalt, Keller, Haass, Thiele, & Simons, 2003; Hattori et al., 2006; Riddell, Christie, Hussain, & Dingwall, 2001). By contrast, acute cell exposure to cholesterol promotes the coclustering of APP and BACE1 in lipid raft domains, as well as their rapid endocytosis (Marquer et al., 2011). Introduction of a glycosylphosphatidylinositol anchor, a targeting motif for lipid raft localization, into the BACE1 sequence strongly promotes amyloidogenic processing of APP (Hartmann et al., 2007; Simons et al., 1998), further supporting the key role of the BACE1 association in Aβ generation. In conclusion, these studies suggest that cholesterol plays an important role in Aβ production by altering the levels of BACE1 in lipid rafts. Beyond cholesterol, some other lipids such as sphingolipids, isoprenoids, and phospholipids also play important roles in Aβ production (Hannun & Obeid, 2008; Hooff, Wood, Muller, & Eckert, 2010; Petanceska & Gandy, 1999).

## 6 CONCLUDING REMARKS

From the above comparison and association studies, we find that AD, in many ways—but not all—accelerated aging. At the molecular level, GWAS of AD and aging hit the same gene, APOE, with the same effect on the two different alleles. The aging field could actually learn from the AD field; wherein, better diagnoses have led to more robust GWAS results, for example, biological age is not taken into consideration for aging GWAS and lifestyle and environment causes need to be controlled or stratified for more repeatable result. DNA methylation and histone modifications show association with aging and AD. Specifically, HDAC inhibition could reverse the cognitive deficits in AD. Noncoding RNA and RNA modifications emerged as a new research focus in aging and AD; so far, no miRNA and lncRNA changes are known to overlap between aging and AD, while changes in RNA editing show a similar pattern in the aging and AD (overall editing levels decrease in both cases).

The cell type composition changes in aging and AD highlight systems level alterations in AD and aging. A significant difference between aging and AD is that the number of neurons does not change very much during aging, but neuron and synapse loss is a hallmark of AD. Microglia gained much attention due to new highly convincing GWAS, which included the association to TREM and CD33. Overall, for glia cell types, there is a proinflammatory environment promoted by aging, which is much more exacerbated in AD.

The surveys at the system levels are still lagging behind, but the circulatory system and the “brain‐gut axis” provide links between the brain and other parts of the body as illustrated by the fascinating example that mice growing under germ‐free conditions show low inflammation level and reduced cerebral Aβ. Overall, chronic inflammation seems to a strong commonality between aging and AD, and if it could be precisely controlled from either the aging or AD perspective based on research products from either field, such treatment could benefit both the aging and AD process and transform the overall landscape of aging and AD.

Among many lifestyle associations and interventions for aging and AD, sleep is clearly strongly associated with brain aging and AD, while the benefits of exercise are still controversial. Metabolic changes in AD are pervasive; however, it converges with aging on insulin and lipid changes; in particular, cholesterol is positively correlated with aging and clearly related to AD pathology.

In summary, aging and AD research are two fields that are intrinsically linked (as summarized in Table 1). Many interventions of aging, such as exercise and calorie restriction (Gunn‐Moore, Kaidanovich‐Beilin, Gallego Iradi, Gunn‐Moore, & Lovestone, 2018), can alleviate AD phenotypes. Drugs and treatments for AD can also slow down aging phenotypes, such as treatments with HDAC inhibitors and neural stem cell transplantation. Although these interventions are validated in mouse models so far, more evidence is in urgent need in human or other primates.

**Table 1:** Summary of commonalities and differences between aging and Alzheimer's disease (AD)

	AD	Aging	Common
Molecular link between aging and AD			
Genetic susceptibility	APP, PSEN1, PSEN2, TREM, CD33, CD1	FOXA3A	APOE
DNA methylation	Epigenetic clock	Association of 5mC and 5nmC	Overexpression of Dnmt3a2 improves the cognitive abilities of aged mice
Histone modifications	HDACi reverses cognitive defects	Loss of repressive marks; gain of activating marks	Acetylation
RNA	Diagnostic tools; BACE1‐AS and BC200	lin‐4 and Dicer	RNA editing decrease
Cell type composition of aging and AD			
Neurons	Marked by synaptic loss	No obvious neuron loss	
Glia	Reactive astrogliosis; microglia pathogenesis	Proinflammatory phenotypes; microglial dystrophy	Proinflammatory environment
Neural stem cells	Olfactory deficits; slow progression	Stem cell capacity loss; extend lifespan	NSC implantation
Systems level of aging and AD			
Systemic inflammation	Viral mimic induction; obesity association	Inflammaging	Strong association
Cardiovascular system	Midlife vascular risk association	Endothelial dysfunction; central arterial stiffness	
	Antibiotic treatment; dental status	Decreased gut microbiome diversity	Germ‐free condition control inflammaging or AD phenotype
Lifestyle and intervention of aging and AD			
Sleep			Sleep apnea; insomnia; NREM
Metabolic	Glucagon‐like peptide 1; phospholipids; leptin; sphingolipids; cholesterol; isoprenoids	Adiponectin; mitochondria	Insulin
Exercise			Cognitive stimulation; physical activity

## References

[acel12802-bib-0001] Ahlskog, J. E. , Geda, Y. E. , Graff‐Radford, N. R. , & Petersen, R. C. (2011). Physical exercise as a preventive or disease‐modifying treatment of dementia and brain aging. Mayo Clinic Proceedings, 86, 876–884. 10.4065/mcp.2011.0252 21878600 PMC3258000

[acel12802-bib-0002] Almond, M. H. , Edwards, M. R. , Barclay, W. S. , & Johnston, S. L. (2013). Obesity and susceptibility to severe outcomes following respiratory viral infection. Thorax, 68, 684–686. 10.1136/thoraxjnl-2012-203009 23436045

[acel12802-bib-0003] Ancoli‐Israel, S. , Klauber, M. R. , Butters, N. , Parker, L. , & Kripke, D. F. (1991). Dementia in institutionalized elderly: Relation to sleep apnea. Journal of the American Geriatrics Society, 39, 258–263. 10.1111/j.1532-5415.1991.tb01647.x 2005339

[acel12802-bib-0004] Ancoli‐Israel, S. , Palmer, B. W. , Cooke, J. R. , Corey‐Bloom, J. , Fiorentino, L. , Natarajan, L. , … Loredo, J. S. (2008). Cognitive effects of treating obstructive sleep apnea in Alzheimer's disease: A randomized controlled study. Journal of the American Geriatrics Society, 56, 2076–2081. 10.1111/j.1532-5415.2008.01934.x 18795985 PMC2585146

[acel12802-bib-0005] Anderson‐Hanley, C. , Arciero, P. J. , Brickman, A. M. , Nimon, J. P. , Okuma, N. , Westen, S. C. , … Zimmerman, E. A. (2012). Exergaming and older adult cognition: A cluster randomized clinical trial. American Journal of Preventive Medicine, 42, 109–119. 10.1016/j.amepre.2011.10.016 22261206

[acel12802-bib-0006] Anselmi, C. V. , Malovini, A. , Roncarati, R. , Novelli, V. , Villa, F. , Condorelli, G. , … Puca, A. A. (2009). Association of the FOXO3A locus with extreme longevity in a southern Italian centenarian study. Rejuvenation Research, 12, 95–104. 10.1089/rej.2008.0827 19415983

[acel12802-bib-0007] Arnold, S. E. , Arvanitakis, Z. , Macauley‐Rambach, S. L. , Koenig, A. M. , Wang, H. Y. , Ahima, R. S. , … Nathan, D. M. (2018). Brain insulin resistance in type 2 diabetes and Alzheimer disease: Concepts and conundrums. NatureReviews Neurology, 14, 168–181. 10.1038/nrneurol.2017.185 29377010 PMC6098968

[acel12802-bib-0008] Barau, J. , Teissandier, A. , Zamudio, N. , Roy, S. , Nalesso, V. , Hérault, Y. , … Bourc'his, D. (2016). The DNA methyltransferase DNMT3C protects male germ cells from transposon activity. Science (New York, NY), 354, 909–912. 10.1126/science.aah5143 27856912

[acel12802-bib-0009] Barnes, D. E. , Santos‐Modesitt, W. , Poelke, G. , Kramer, A. F. , Castro, C. , Middleton, L. E. , & Yaffe, K. (2013). The Mental Activity and eXercise (MAX) trial: A randomized controlled trial to enhance cognitive function in older adults. Jamainternal Medicine, 173, 797–804. 10.1001/jamainternmed.2013.189 PMC5921904 23545598

[acel12802-bib-0010] Barzilai, N. , Huffman, D. M. , Muzumdar, R. H. , & Bartke, A. (2012). The critical role of metabolic pathways in aging. Diabetes, 61, 1315–1322. 10.2337/db11-1300 22618766 PMC3357299

[acel12802-bib-0011] Bastard, J.‐P. , Maachi, M. , Lagathu, C. , Kim, M. J. , Caron, M. , Vidal, H. , … Feve, B. (2006). Recent advances in the relationship between obesity, inflammation, and insulin resistance. European Cytokine Network, 17, 4–12. 16613757

[acel12802-bib-0012] Benito, E. , Urbanke, H. , Ramachandran, B. , Barth, J. , Halder, R. , Awasthi, A. , … Fischer, A. (2015). HDAC inhibitor‐dependent transcriptome and memory reinstatement in cognitive decline models. Journal of Clinical Investigation, 125, 3572–3584. 10.1172/JCI79942 26280576 PMC4588238

[acel12802-bib-0013] Berg, A. H. , & Scherer, P. E. (2005). Adipose tissue, inflammation, and cardiovascular disease. Circulation Research, 96, 939–949. 10.1161/01.RES.0000163635.62927.34 15890981

[acel12802-bib-0014] Bhattacharyya, R. , & Kovacs, D. M. (2010). ACAT inhibition and amyloid beta reduction. Biochimica Et Biophysica Acta, 1801, 960–965. 10.1016/j.bbalip.2010.04.003 20398792 PMC2918257

[acel12802-bib-0015] Bjorklund, N. L. , Reese, L. C. , Sadagoparamanujam, V. M. , Ghirardi, V. , Woltjer, R. L. , & Taglialatela, G. (2012). Absence of amyloid β oligomers at the postsynapse and regulated synaptic Zn2+ in cognitively intact aged individuals with Alzheimer's disease neuropathology. Molecular Neurodegeneration, 7, 23 10.1186/1750-1326-7-23 22640423 PMC3403985

[acel12802-bib-0016] Blurton‐Jones, M. , Kitazawa, M. , Martinez‐Coria, H. , Castello, N. A. , Müller, F.‐J. , Loring, J. F. , … LaFerla, F. M. (2009). Neural stem cells improve cognition via BDNF in a transgenic model of Alzheimer disease. Proceedings of the National Academy of Sciences of the United States of America, 106, 13594–13599. 10.1073/pnas.0901402106 19633196 PMC2715325

[acel12802-bib-0017] Boldrini, M. , Fulmore, C. A. , Tartt, A. N. , Simeon, L. R. , Pavlova, I. , Poposka, V. , … Mann, J. J. (2018). Human hippocampal neurogenesis persists throughout aging. CellStem Cell, 22, 589–599.e585. 10.1016/j.stem.2018.03.015 PMC5957089 29625071

[acel12802-bib-0018] Booth, L. N. , & Brunet, A. (2016). The aging epigenome. Molecular Cell, 62, 728–744. 10.1016/j.molcel.2016.05.013 27259204 PMC4917370

[acel12802-bib-0019] Braak, H. , & Tredici, K. D. (2011). The pathological process underlying Alzheimer’s disease in individuals under thirty. Acta Neuropathologica, 121, 171–181. 10.1007/s00401-010-0789-4 21170538

[acel12802-bib-0020] Braun, A. R. , Balkin, T. J. , Wesenten, N. J. , Carson, R. E. , Varga, M. , Baldwin, P. , … Herscovitch, P. (1997). Regional cerebral blood flow throughout the sleep‐wake cycle. An H2(15)O PET study. Brain, 120, 1173–1197. 10.1093/brain/120.7.1173 9236630

[acel12802-bib-0021] Buchsbaum, M. S. , Gillin, J. C. , Wu, J. , Hazlett, E. , Sicotte, N. , Dupont, R. M. , & Bunney, W. E. (1989). Regional cerebral glucose metabolic rate in human sleep assessed by positron emission tomography. Life Sciences, 45, 1349–1356. 10.1016/0024-3205(89)90021-0 2796606

[acel12802-bib-0022] Burke, S. N. , & Barnes, C. A. (2006). Neural plasticity in the ageing brain. Nature Reviews Neuroscience, 7(1), 30–40. 10.1038/nrn1809 16371948

[acel12802-bib-0023] Celermajer, D. S. , Sorensen, K. E. , Spiegelhalter, D. J. , Georgakopoulos, D. , Robinson, J. , & Deanfield, J. E. (1994). Aging is associated with endothelial dysfunction in healthy men years before the age‐related decline in women. Journal of the American College of Cardiology, 24, 471–476. 10.1016/0735-1097(94)90305-0 8034885

[acel12802-bib-0024] Chang, T. Y. , Chang, C. C. , Ohgami, N. , & Yamauchi, Y. (2006). Cholesterol sensing, trafficking, and esterification. Annual Review of Cell and Developmental Biology, 22, 129–157. 10.1146/annurev.cellbio.22.010305.104656 16753029

[acel12802-bib-0025] Cockburn, A. F. , Dehlin, J. M. , Ngan, T. , Crout, R. , Boskovic, G. , Denvir, J. , … Cuff, C. F. (2012). High throughput DNA sequencing to detect differences in the subgingival plaque microbiome in elderly subjects with and without dementia. Investigative Genetics, 3, 19 10.1186/2041-2223-3-19 22998923 PMC3488532

[acel12802-bib-0026] De Jager, P. L. , Srivastava, G. , Lunnon, K. , Burgess, J. , Schalkwyk, L. C. , Yu, L. , … Bennett, D. A. (2014). Alzheimer's disease: Early alterations in brain DNA methylation at ANK1, BIN1, RHBDF2 and other loci. Nature Neuroscience, 17, 1156–1163. 10.1038/nn.3786 25129075 PMC4292795

[acel12802-bib-0027] De Strooper, B. , & Karran, E. (2016). The cellular phase of Alzheimer’s disease. Cell, 164, 603–615. 10.1016/j.cell.2015.12.056 26871627

[acel12802-bib-0028] Deelen, J. , Beekman, M. , Uh, H.‐W. , Helmer, Q. , Kuningas, M. , Christiansen, L. , … Slagboom, P. E. (2011). Genome‐wide association study identifies a single major locus contributing to survival into old age; the APOE locus revisited. Aging Cell, 10, 686–698. 10.1111/j.1474-9726.2011.00705.x 21418511 PMC3193372

[acel12802-bib-0029] Devanand, D. P. , Michaels‐Marston, K. S. , Liu, X. , Pelton, G. H. , Padilla, M. , Marder, K. , … Mayeux, R. (2000). Olfactory deficits in patients with mild cognitive impairment predict Alzheimer's disease at follow‐up. American Journal of Psychiatry, 157, 1399–1405. 10.1176/appi.ajp.157.9.1399 10964854

[acel12802-bib-0030] Dietschy, J. M. , & Turley, S. D. (2001). Cholesterol metabolism in the brain. Current Opinion in Lipidology, 12, 105–112. 10.1097/00041433-200104000-00003 11264981

[acel12802-bib-0031] Dirks, A. J. , Hofer, T. , Marzetti, E. , Pahor, M. , & Leeuwenburgh, C. (2006). Mitochondrial DNA mutations, energy metabolism and apoptosis in aging muscle. Ageing Research Reviews, 5, 179–195. 10.1016/j.arr.2006.03.002 16647308

[acel12802-bib-0032] dos Santos Moraes, W. A. , Poyares, D. R. , Guilleminault, C. , Ramos, L. R. , Bertolucci, P. H. F. , & Tufik, S. (2006). The effect of Donepezil on sleep and REM sleep EEG in patients with Alzheimer disease: A double‐blind placebo‐controlled study. Sleep, 29, 199–205. 10.1093/sleep/29.2.199 16494088

[acel12802-bib-0033] Duzel, E. , van Praag, H. , & Sendtner, M. (2016). Can physical exercise in old age improve memory and hippocampal function? Brain, 139, 662–673. 10.1093/brain/awv407 26912638 PMC4766381

[acel12802-bib-0034] Dworak, M. , McCarley, R. W. , Kim, T. , Kalinchuk, A. V. , & Basheer, R. (2010). Sleep and brain energy levels: ATP changes during sleep. The Journal of Neuroscience, 30, 9007–9016. 10.1523/JNEUROSCI.1423-10.2010 20592221 PMC2917728

[acel12802-bib-0035] Ehehalt, R. , Keller, P. , Haass, C. , Thiele, C. , & Simons, K. (2003). Amyloidogenic processing of the Alzheimer beta‐amyloid precursor protein depends on lipid rafts. Journal of Cell Biology, 160, 113–123. 10.1083/jcb.200207113 12515826 PMC2172747

[acel12802-bib-0036] Everson, C. A. , Henchen, C. J. , Szabo, A. , & Hogg, N. (2014). Cell injury and repair resulting from sleep loss and sleep recovery in laboratory rats. Sleep, 37, 1929–1940. 10.5665/sleep.4244 25325492 PMC4548518

[acel12802-bib-0037] Ewbank, D. C. (2007). Differences in the association between apolipoprotein E genotype and mortality across populations. Journals of Gerontology. Series A, Biological Sciences and Medical Sciences, 62, 899–907. 10.1093/gerona/62.8.899 17702883

[acel12802-bib-0038] Fabricius, K. , Jacobsen, J. S. , & Pakkenberg, B. (2013). Effect of age on neocortical brain cells in 90+ year old human females–a cell counting study. Neurobiology of Aging, 34, 91–99. 10.1016/j.neurobiolaging.2012.06.009 22878165

[acel12802-bib-0039] Fan, X. , Wheatley, E. G. , & Villeda, S. A. (2017). Mechanisms of hippocampal aging and the potential for rejuvenation. Annual Review of Neuroscience, 40, 251–272. 10.1146/annurev-neuro-072116-031357 28441118

[acel12802-bib-0040] Fassbender, K. , Simons, M. , Bergmann, C. , Stroick, M. , Lütjohann, D. , Keller, P. , … Hartmann, T. (2001). Simvastatin strongly reduces levels of Alzheimer's disease β‐amyloid peptides Aβ42 and Aβ40 in vitro and in vivo. Proceedings of the National Academy of Sciences of the United States of America, 98, 5856–5861. 10.1073/pnas.081620098 11296263 PMC33303

[acel12802-bib-0041] Feser, J. , Truong, D. , Das, C. , Carson, J. J. , Kieft, J. , Harkness, T. , & Tyler, J. K. (2010). Elevated histone expression promotes life span extension. Molecular Cell, 39, 724–735. 10.1016/j.molcel.2010.08.015 20832724 PMC3966075

[acel12802-bib-0042] Flachsbart, F. , Caliebe, A. , Kleindorp, R. , Blanché, H. , von Eller‐Eberstein, H. , Nikolaus, S. , … Nebel, A. (2009). Association of FOXO3A variation with human longevity confirmed in German centenarians. Proceedings of the National Academy of Sciences of the United States of America, 106, 2700–2705. 10.1073/pnas.0809594106 19196970 PMC2650329

[acel12802-bib-0043] Foley, P. (2010). Lipids in Alzheimer's disease: A century‐old story. Biochimica Et Biophysica Acta (BBA) ‐ Molecular and Cell Biology of Lipids, 1801, 750–753. 10.1016/j.bbalip.2010.05.004 20471492

[acel12802-bib-0044] Franceschi, C. , Garagnani, P. , Vitale, G. , Capri, M. , & Salvioli, S. (2017). Inflammaging and ‘Garb‐aging’. Trends in Endocrinology & Metabolism, 28, 199–212. 10.1016/j.tem.2016.09.005 27789101

[acel12802-bib-0045] Fritze, O. , Romero, B. , Schleicher, M. , Jacob, M. P. , Oh, D.‐Y. , Starcher, B. , … Stock, U. A. (2012). Age‐related changes in the elastic tissue of the human aorta. Journal of Vascular Research, 49, 77–86. 10.1159/000331278 22105095

[acel12802-bib-0046] Frolich, L. , Blum‐Degen, D. , Bernstein, H. G. , Engelsberger, S. , Humrich, J. , Laufer, S. , … Riederer, P. (1998). Brain insulin and insulin receptors in aging and sporadic Alzheimer's disease. Journal of Neural Transmission (Vienna, Austria: 1996), 105, 423–438. 10.1007/s007020050068 9720972

[acel12802-bib-0047] Funayama, R. , Saito, M. , Tanobe, H. , & Ishikawa, F. (2006). Loss of linker histone H1 in cellular senescence. Journal of Cell Biology, 175, 869–880. 10.1083/jcb.200604005 17158953 PMC2064697

[acel12802-bib-0048] Gaisler‐Salomon, I. , Kravitz, E. , Feiler, Y. , Safran, M. , Biegon, A. , Amariglio, N. , & Rechavi, G. (2014). Hippocampus‐specific deficiency in RNA editing of GluA2 in Alzheimer's disease. Neurobiology of Aging, 35, 1785–1791. 10.1016/j.neurobiolaging.2014.02.018 24679603

[acel12802-bib-0049] Gatz, M. , Mortimer, J. A. , Fratiglioni, L. , Johansson, B. , Berg, S. , Reynolds, C. A. , & Pedersen, N. L. (2006). Potentially modifiable risk factors for dementia in identical twins. Alzheimer's & Dementia: the Journal of the Alzheimer's Association, 2, 110–117. 10.1016/j.jalz.2006.01.002 19595867

[acel12802-bib-0050] Geda, Y. E. , Silber, T. C. , Roberts, R. O. , Knopman, D. S. , Christianson, T. J. , Pankratz, V. S. , … Petersen, R. C. (2012). Computer activities, physical exercise, aging, and mild cognitive impairment: A population‐based study. Mayo Clinic Proceedings, 87, 437–442. 10.1016/j.mayocp.2011.12.020 22560523 PMC3538471

[acel12802-bib-0051] Gerdes, L. U. , Jeune, B. , Ranberg, K. A. , Nybo, H. , & Vaupel, J. W. (2000). Estimation of apolipoprotein E genotype‐specific relative mortality risks from the distribution of genotypes in centenarians and middle‐aged men: Apolipoprotein E gene is a "frailty gene," not a "longevity gene". Genetic Epidemiology, 19, 202–210. 10.1002/1098-2272(200010)19:3xxaaa202:AID-GEPI2xxbbb3.0.CO;2-Q 11015124

[acel12802-bib-0052] Gottesman, R. F. , Schneider, A. L. C. , Zhou, Y. , Coresh, J. , Green, E. , Gupta, N. , … Mosley, T. H. (2017). Association between midlife vascular risk factors and estimated brain amyloid deposition. JAMA, 317, 1443–1450. 10.1001/jama.2017.3090 28399252 PMC5921896

[acel12802-bib-0053] Grabert, K. , Michoel, T. , Karavolos, M. H. , Clohisey, S. , Baillie, J. K. , Stevens, M. P. , … McColl, B. W. (2016). Microglial brain region‐dependent diversity and selective regional sensitivities to aging. Nature Neuroscience, 19, 504–516. 10.1038/nn.4222 26780511 PMC4768346

[acel12802-bib-0054] Gräff, J. , Rei, D. , Guan, J.‐S. , Wang, W.‐Y. , Seo, J. , Hennig, K. M. , … Tsai, L.‐H. (2012). An epigenetic blockade of cognitive functions in the neurodegenerating brain. Nature, 483, 222–226. 10.1038/nature10849 22388814 PMC3498952

[acel12802-bib-0055] Grothe, M. J. , Villeneuve, S. , Dyrba, M. , Bartrés‐Faz, D. , Wirth, M. , Neuroimaging, A. D. , & Alzheimer's Disease Neuroimaging, I. (2017). Multimodal characterization of older APOE2 carriers reveals selective reduction of amyloid load. Neurology, 88, 569–576. 10.1212/WNL.0000000000003585 28062720 PMC5304459

[acel12802-bib-0056] Guarnieri, B. , Adorni, F. , Musicco, M. , Appollonio, I. , Bonanni, E. , Caffarra, P. , … Sorbi, S. (2012). Prevalence of sleep disturbances in mild cognitive impairment and dementing disorders: A multicenter italian clinical cross‐sectional study on 431 patients. Dementia and Geriatric Cognitive Disorders, 33, 50–58. 10.1159/000335363 22415141 PMC3696366

[acel12802-bib-0057] Guerreiro, R. , Wojtas, A. , Bras, J. , Carrasquillo, M. , Rogaeva, E. , Majounie, E. , … G., (2013). TREM2 variants in Alzheimer's disease. The New England Journal of Medicine, 368, 117–127. 10.1056/NEJMoa1211851 23150934 PMC3631573

[acel12802-bib-0058] Gunn‐Moore, D. , Kaidanovich‐Beilin, O. , Gallego Iradi, M. C. , Gunn‐Moore, F. , & Lovestone, S. (2018). Alzheimer's disease in humans and other animals: A consequence of postreproductive life span and longevity rather than aging. Alzheimer's & Dementia: the Journal of the Alzheimer's Association, 14, 195–204. 10.1016/j.jalz.2017.08.014 28972881

[acel12802-bib-0059] Hannun, Y. A. , & Obeid, L. M. (2008). Principles of bioactive lipid signalling: Lessons from sphingolipids. Nature Reviews Molecular Cell Biology, 9, 139–150. 10.1038/nrm2329 18216770

[acel12802-bib-0060] Harach, T. , Marungruang, N. , Duthilleul, N. , Cheatham, V. , Mc Coy, K. D. , Frisoni, G. , … Bolmont, T. (2017). Reduction of Abeta amyloid pathology in APPPS1 transgenic mice in the absence of gut microbiota. Scientific Reports, 7, 41802 10.1038/srep41802 28176819 PMC5297247

[acel12802-bib-0061] Harry, G. J. (2013). Microglia during development and aging. Pharmacology & Therapeutics, 139, 313–326. 10.1016/j.pharmthera.2013.04.013 23644076 PMC3737416

[acel12802-bib-0062] Hartmann, T. , Kuchenbecker, J. , & Grimm, M. O. (2007). Alzheimer's disease: The lipid connection. Journal of Neurochemistry, 103(Suppl 1), 159–170. 10.1111/j.1471-4159.2007.04715.x 17986151

[acel12802-bib-0063] Hattori, C. , Asai, M. , Onishi, H. , Sasagawa, N. , Hashimoto, Y. , Saido, T. C. , … Ishiura, S. (2006). BACE1 interacts with lipid raft proteins. Journal of Neuroscience Research, 84, 912–917. 10.1002/jnr.20981 16823808

[acel12802-bib-0064] Heneka, M. T. , Carson, M. J. , Khoury, J. E. , Landreth, G. E. , Brosseron, F. , Feinstein, D. L. , … Kummer, M. P. (2015). Neuroinflammation in Alzheimer's disease. The Lancet Neurology, 14, 388–405. 10.1016/S1474-4422(15)70016-5 25792098 PMC5909703

[acel12802-bib-0065] Heppner, F. L. , Ransohoff, R. M. , & Becher, B. (2015). Immune attack: The role of inflammation in Alzheimer disease. Nature Reviews Neuroscience, 16, 358–372. 10.1038/nrn3880 25991443

[acel12802-bib-0066] Heyn, H. , Li, N. , Ferreira, H. J. , Moran, S. , Pisano, D. G. , Gomez, A. , … Esteller, M. (2012). Distinct DNA methylomes of newborns and centenarians. Proceedings of the National Academy of Sciences of the United States of America, 109, 10522–10527. 10.1073/pnas.1120658109 22689993 PMC3387108

[acel12802-bib-0067] Hooff, G. P. , Wood, W. G. , Muller, W. E. , & Eckert, G. P. (2010). Isoprenoids, small GTPases and Alzheimer's disease. Biochimica Et Biophysica Acta, 1801, 896–905. 10.1016/j.bbalip.2010.03.014 20382260 PMC2886181

[acel12802-bib-0068] Hutter‐Paier, B. , Huttunen, H. J. , Puglielli, L. , Eckman, C. B. , Kim, D. Y. , Hofmeister, A. , … Kovacs, D. M. (2004). The ACAT inhibitor CP‐113,818 markedly reduces amyloid pathology in a mouse model of Alzheimer's disease. Neuron, 44, 227–238. 10.1016/j.neuron.2004.08.043 15473963

[acel12802-bib-0069] Ito, S. , Shen, L. , Dai, Q. , Wu, S. C. , Collins, L. B. , Swenberg, J. A. , … Zhang, Y. (2011). Tet proteins can convert 5‐methylcytosine to 5‐formylcytosine and 5‐carboxylcytosine. Science (New York, NY), 333, 1300–1303. 10.1126/science.1210597 PMC3495246 21778364

[acel12802-bib-0070] Jack, C. R. , Knopman, D. S. , Jagust, W. J. , Shaw, L. M. , Aisen, P. S. , Weiner, M. W. , … Trojanowski, J. Q. (2010). Hypothetical model of dynamic biomarkers of the Alzheimer's pathological cascade. The Lancet Neurology, 9, 119–128. 10.1016/S1474-4422(09)70299-6 20083042 PMC2819840

[acel12802-bib-0071] Jackson, M. A. , Jackson, M. , Jeffery, I. B. , Beaumont, M. , Bell, J. T. , Clark, A. G. , … Steves, C. J. (2016). Signatures of early frailty in the gut microbiota. Genome Medicine, 8, 8 10.1186/s13073-016-0262-7 26822992 PMC4731918

[acel12802-bib-0072] Jonsson, T. , Stefansson, H. , Steinberg, S. , Jonsdottir, I. , Jonsson, P. V. , Snaedal, J. , … Stefansson, K. (2013). Variant of TREM2 associated with the risk of Alzheimer's disease. The New England Journal of Medicine, 368, 107–116. 10.1056/NEJMoa1211103 23150908 PMC3677583

[acel12802-bib-0073] Junn, E. , & Mouradian, M. M. (2012). MicroRNAs in neurodegenerative diseases and their therapeutic potential. Pharmacology & Therapeutics, 133, 142–150. 10.1016/j.pharmthera.2011.10.002 22008259 PMC3268953

[acel12802-bib-0074] Kang, J.‐E. , Lim, M. M. , Bateman, R. J. , Lee, J. J. , Smyth, L. P. , Cirrito, J. R. , … Holtzman, D. M. (2009). Amyloid‐β dynamics are regulated by orexin and the sleep‐wake cycle. Science, 326, 1005–1007. 10.1126/science.1180962 19779148 PMC2789838

[acel12802-bib-0075] Karch, C. M. , & Goate, A. M. (2015). Alzheimer’s disease risk genes and mechanisms of disease pathogenesis. Biological Psychiatry, 77, 43–51. 10.1016/j.biopsych.2014.05.006 24951455 PMC4234692

[acel12802-bib-0076] Kato, M. , & Slack, F. J. (2013). Ageing and the small, non‐coding RNA world. Ageing Research Reviews, 12, 429–435. 10.1016/j.arr.2012.03.012 22504407 PMC3405179

[acel12802-bib-0077] Keren‐Shaul, H. , Spinrad, A. , Weiner, A. , Matcovitch‐Natan, O. , Dvir‐Szternfeld, R. , Ulland, T. K. , … Amit, I. (2017). A unique microglia type associated with restricting development of Alzheimer’s disease. Cell, 169(7), 1276–1290.e17. 10.1016/j.cell.2017.05.018 28602351

[acel12802-bib-0078] Khermesh, K. , D'Erchia, A. M. , Barak, M. , Annese, A. , Wachtel, C. , Levanon, E. Y. , … Eisenberg, E. (2016). Reduced levels of protein recoding by A‐to‐I RNA editing in Alzheimer's disease. RNA (New York, NY), 22, 290–302. 10.1261/rna.054627.115 PMC4712678 26655226

[acel12802-bib-0079] Kilgore, M. , Miller, C. A. , Fass, D. M. , Hennig, K. M. , Haggarty, S. J. , Sweatt, J. D. , & Rumbaugh, G. (2010). Inhibitors of class 1 histone deacetylases reverse contextual memory deficits in a mouse model of Alzheimer's disease. Neuropsychopharmacology, 35, 870–880. 10.1038/npp.2009.197 20010553 PMC3055373

[acel12802-bib-0080] Klein, H.‐U. , & De Jager, P. L. (2016). Uncovering the role of the methylome in dementia and neurodegeneration. Trends in Molecular Medicine, 22, 687–700. 10.1016/j.molmed.2016.06.008 27423266

[acel12802-bib-0081] Kour, S. , & Rath, P. C. (2016). Long noncoding RNAs in aging and age‐related diseases. Ageing Research Reviews, 26, 1–21. 10.1016/j.arr.2015.12.001 26655093

[acel12802-bib-0082] Krabbe, G. , Halle, A. , Matyash, V. , Rinnenthal, J. L. , Eom, G. D. , Bernhardt, U. , … Heppner, F. L. (2013). Functional impairment of microglia coincides with Beta‐amyloid deposition in mice with Alzheimer‐like pathology. PloS One, 8, e60921 10.1371/journal.pone.0060921 23577177 PMC3620049

[acel12802-bib-0083] Krstic, D. , Madhusudan, A. , Doehner, J. , Vogel, P. , Notter, T. , Imhof, C. , … Knuesel, I. (2012). Systemic immune challenges trigger and drive Alzheimer‐like neuropathology in mice. Journal of Neuroinflammation, 9, 151 10.1186/1742-2094-9-151 22747753 PMC3483167

[acel12802-bib-0084] Lam, Q. L. , & Lu, L. (2007). Role of leptin in immunity. Cellular & Molecular Immunology, 4, 1–13. 17349207

[acel12802-bib-0085] Lambert, J. C. , Ibrahim‐Verbaas, C. A. , Harold, D. , Naj, A. C. , Sims, R. , Bellenguez, C. , … Amouyel, P. (2013). Meta‐analysis of 74,046 individuals identifies 11 new susceptibility loci for Alzheimer's disease. Nature Genetics, 45, 1452–1458. 10.1038/ng.2802 24162737 PMC3896259

[acel12802-bib-0086] Lardenoije, R. , Iatrou, A. , Kenis, G. , Kompotis, K. , Steinbusch, H. W. M. , Mastroeni, D. , … Rutten, B. P. F. (2015). The epigenetics of aging and neurodegeneration. Progress in Neurobiology, 131, 21–64. 10.1016/j.pneurobio.2015.05.002 26072273 PMC6477921

[acel12802-bib-0087] Levine, M. E. , Lu, A. T. , Bennett, D. A. , & Horvath, S. (2015). Epigenetic age of the pre‐frontal cortex is associated with neuritic plaques, amyloid load, and Alzheimer's disease related cognitive functioning. Aging (Albany NY), 7, 1198–1211. https://doi.org/10.18632/aging.100864 26684672 10.18632/aging.100864 PMC4712342

[acel12802-bib-0088] Li, X. L. , Aou, S. , Oomura, Y. , Hori, N. , Fukunaga, K. , & Hori, T. (2002). Impairment of long‐term potentiation and spatial memory in leptin receptor‐deficient rodents. Neuroscience, 113, 607–615. 10.1016/S0306-4522(02)00162-8 12150780

[acel12802-bib-0089] Li, S. , Jin, M. , Zhang, D. , Yang, T. , Koeglsperger, T. , Fu, H. , & Selkoe, D. J. (2013). Environmental novelty activates beta2‐adrenergic signaling to prevent the impairment of hippocampal LTP by Abeta oligomers. Neuron, 77, 929–941. 10.1016/j.neuron.2012.12.040 23473322 PMC3596823

[acel12802-bib-0090] Li, Y. , Wang, W.‐J. , Cao, H. , Lu, J. , Wu, C. , Hu, F.‐Y. , … Tian, X.‐L. (2009). Genetic association of FOXO1A and FOXO3A with longevity trait in Han Chinese populations. Human Molecular Genetics, 18, 4897–4904. 10.1093/hmg/ddp459 19793722 PMC2790334

[acel12802-bib-0091] Liddelow, S. A. , & Barres, B. A. (2017). Reactive astrocytes: production, function, and therapeutic potential. Immunity, 46, 957–967. 10.1016/j.immuni.2017.06.006 28636962

[acel12802-bib-0092] Lieb, W. , Beiser, A. S. , Vasan, R. S. , Tan, Z. S. , Au, R. , Harris, T. B. , … Seshadri, S. (2009). Association of plasma leptin levels with incident Alzheimer disease and MRI measures of brain aging. JAMA, 302, 2565–2572. 10.1001/jama.2009.1836 20009056 PMC2838501

[acel12802-bib-0093] Liguori, C. , Romigi, A. , Nuccetelli, M. , Zannino, S. , Sancesario, G. , Martorana, A. , … Placidi, F. (2014). Orexinergic system dysregulation, sleep impairment, and cognitive decline in alzheimer disease. JAMA Neurology, 71, 1498–1505. 10.1001/jamaneurol.2014.2510 25322206

[acel12802-bib-0094] Limke, T. L. , & Rao, M. S. (2002). Neural stem cells in aging and disease. Journal of Cellular and Molecular Medicine, 6, 475–496. 10.1111/j.1582-4934.2002.tb00451.x 12611637 PMC6741307

[acel12802-bib-0095] Lovden, M. , Schaefer, S. , Noack, H. , Bodammer, N. C. , Kuhn, S. , Heinze, H. J. , … Lindenberger, U. (2012). Spatial navigation training protects the hippocampus against age‐related changes during early and late adulthood. Neurobiology of Aging, 33, 620.e629–620.e622. 10.1016/j.neurobiolaging.2011.02.013 21497950

[acel12802-bib-0096] Lunnon, K. , Smith, R. , Hannon, E. , De Jager, P. L. , Srivastava, G. , Volta, M. , … Mill, J. (2014). Methylomic profiling implicates cortical deregulation of ANK1 in Alzheimer's disease. Nature Neuroscience, 17, 1164–1170. 10.1038/nn.3782 25129077 PMC4410018

[acel12802-bib-0097] Luo, Q. , & Chen, Y. (2016). Long noncoding RNAs and Alzheimer's disease. Clinical Interventions in Aging, 11, 867‐872. 27418812 10.2147/CIA.S107037 PMC4933566

[acel12802-bib-0098] Marquer, C. , Devauges, V. , Cossec, J. C. , Liot, G. , Lecart, S. , Saudou, F. , … Potier, M. C. (2011). Local cholesterol increase triggers amyloid precursor protein‐Bace1 clustering in lipid rafts and rapid endocytosis. The FASEB Journal, 25, 1295–1305. 10.1096/fj.10-168633 21257714

[acel12802-bib-0099] Martin, B. , Golden, E. , Carlson, O. D. , Egan, J. M. , Mattson, M. P. , & Maudsley, S. (2008). Caloric restriction: Impact upon pituitary function and reproduction. Ageing Research Reviews, 7, 209–224. 10.1016/j.arr.2008.01.002 18329344 PMC2634963

[acel12802-bib-0100] Martin, B. , Golden, E. , Egan, J. M. , Mattson, M. P. , & Maudsley, S. (2007). Reduced energy intake: The secret to a long and healthy life? IBS Journal of Science, 2, 35–39. 18985162 PMC2577199

[acel12802-bib-0101] Martin, B. , Ji, S. , Maudsley, S. , & Mattson, M. P. (2010). "Control" laboratory rodents are metabolically morbid: Why it matters. Proceedings of the National Academy of Sciences of the United States of America, 107, 6127–6133. 10.1073/pnas.0912955107 20194732 PMC2852022

[acel12802-bib-0102] Martin, B. , Mattson, M. P. , & Maudsley, S. (2006). Caloric restriction and intermittent fasting: Two potential diets for successful brain aging. Ageing Research Reviews, 5, 332–353. 10.1016/j.arr.2006.04.002 16899414 PMC2622429

[acel12802-bib-0103] Massaad, C. A. , & Klann, E. (2011). Reactive oxygen species in the regulation of synaptic plasticity and memory. Antioxidants & Redox Signaling, 14, 2013–2054. 10.1089/ars.2010.3208 20649473 PMC3078504

[acel12802-bib-0104] Mattick, J. S. , & Rinn, J. L. (2015). Discovery and annotation of long noncoding RNAs. Nature Structural & Molecular Biology, 22, 5–7. 10.1038/nsmb.2942 25565026

[acel12802-bib-0105] Menkes‐Caspi, N. , Yamin, H. G. , Kellner, V. , Spires‐Jones, T. L. , Cohen, D. , & Stern, E. A. (2015). Pathological tau disrupts ongoing network activity. Neuron, 85, 959–966. 10.1016/j.neuron.2015.01.025 25704951

[acel12802-bib-0106] Millan, M. J. (2017). Linking deregulation of non‐coding RNA to the core pathophysiology of Alzheimer’s disease: An integrative review. Progress in Neurobiology, 156, 1–68. 10.1016/j.pneurobio.2017.03.004 28322921

[acel12802-bib-0107] Minter, M. R. , Zhang, C. , Leone, V. , Ringus, D. L. , Zhang, X. , Oyler‐Castrillo, P. , … Sisodia, S. S. (2016). Antibiotic‐induced perturbations in gut microbial diversity influences neuro‐inflammation and amyloidosis in a murine model of Alzheimer's disease. Scientific Reports, 6, 30028 10.1038/srep30028 27443609 PMC4956742

[acel12802-bib-0108] Misonou, H. , Morishima‐Kawashima, M. , & Ihara, Y. (2000). Oxidative stress induces intracellular accumulation of amyloid β‐protein (Aβ) in human neuroblastoma cells. Biochemistry, 39, 6951–6959. 10.1021/bi000169p 10841777

[acel12802-bib-0109] Montaron, M. F. , Petry, K. G. , Rodriguez, J. J. , Marinelli, M. , Aurousseau, C. , Rougon, G. , … Abrous, D. N. (1999). Adrenalectomy increases neurogenesis but not PSA‐NCAM expression in aged dentate gyrus. European Journal of Neuroscience, 11, 1479–1485. 10.1046/j.1460-9568.1999.00579.x 10103142

[acel12802-bib-0110] Morley, J. E. (2008). Diabetes and aging: Epidemiologic overview. Clinics in Geriatric Medicine, 24(3), 395–405. 10.1016/j.cger.2008.03.005 18672179

[acel12802-bib-0111] Mosher, K. I. , & Wyss‐Coray, T. (2014). Microglial dysfunction in brain aging and Alzheimer's disease. Biochemical Pharmacology, 88, 594–604. 10.1016/j.bcp.2014.01.008 24445162 PMC3972294

[acel12802-bib-0112] Ngandu, T. , Lehtisalo, J. , Solomon, A. , Levalahti, E. , Ahtiluoto, S. , Antikainen, R. , … Kivipelto, M. (2015). A 2 year multidomain intervention of diet, exercise, cognitive training, and vascular risk monitoring versus control to prevent cognitive decline in at‐risk elderly people (FINGER): A randomised controlled trial. Lancet, 385, 2255–2263. 10.1016/S0140-6736(15)60461-5 25771249

[acel12802-bib-0113] Nishikura, K. (2016). A‐to‐I editing of coding and non‐coding RNAs by ADARs. Nature Reviews Molecular Cell Biology, 17, 83–96. 10.1038/nrm.2015.4 26648264 PMC4824625

[acel12802-bib-0114] Norton, S. , Matthews, F. E. , Barnes, D. E. , Yaffe, K. , & Brayne, C. (2014). Potential for primary prevention of Alzheimer's disease: An analysis of population‐based data. The Lancet Neurology, 13, 788–794. 10.1016/S1474-4422(14)70136-X 25030513

[acel12802-bib-0115] Oh, G. , Ebrahimi, S. , Wang, S.‐C. , Cortese, R. , Kaminsky, Z. A. , Gottesman, I. I. , … Petronis, A. (2016). Epigenetic assimilation in the aging human brain. Genome Biology, 17, 76 10.1186/s13059-016-0946-8 27122015 PMC4848814

[acel12802-bib-0116] Okonkwo, O. C. , Schultz, S. A. , Oh, J. M. , Larson, J. , Edwards, D. , Cook, D. , … Sager, M. A. (2014). Physical activity attenuates age‐related biomarker alterations in preclinical AD. Neurology, 83, 1753–1760. 10.1212/WNL.0000000000000964 25298312 PMC4239838

[acel12802-bib-0117] Oliveira, A. M. M. , Hemstedt, T. J. , & Bading, H. (2012). Rescue of aging‐associated decline in Dnmt3a2 expression restores cognitive abilities. Nature Neuroscience, 15, 1111–1113. 10.1038/nn.3151 22751036

[acel12802-bib-0118] Osawa, S. , Funamoto, S. , Nobuhara, M. , Wada‐Kakuda, S. , Shimojo, M. , Yagishita, S. , & Ihara, Y. (2008). Phosphoinositides suppress gamma‐secretase in both the detergent‐soluble and ‐insoluble states. Journal of Biological Chemistry, 283, 19283–19292. 10.1074/jbc.M705954200 18480063

[acel12802-bib-0119] Osborn, L. M. , Kamphuis, W. , Wadman, W. J. , & Hol, E. M. (2016). Astrogliosis: An integral player in the pathogenesis of Alzheimer's disease. Progress in Neurobiology, 144, 121–141. 10.1016/j.pneurobio.2016.01.001 26797041

[acel12802-bib-0120] Osenkowski, P. , Ye, W. , Wang, R. , Wolfe, M. S. , & Selkoe, D. J. (2008). Direct and potent regulation of gamma‐secretase by its lipid microenvironment. Journal of Biological Chemistry, 283, 22529–22540. 10.1074/jbc.M801925200 18539594 PMC2504869

[acel12802-bib-0121] Osorio, R. S. , Gumb, T. , Pirraglia, E. , Varga, A. W. , Lu, S.‐E. , Lim, J. , … For the Alzheimer's Disease Neuroimaging InitiativeWeinerMichael WmdaisenPaulMdpetersenRonaldMd, P.C.J.Q.M.D.P.W. (2015). Sleep‐disordered breathing advances cognitive decline in the elderly. Neurology, 84, 1964–1971. 10.1212/WNL.0000000000001566 25878183 PMC4433459

[acel12802-bib-0122] Osorio, R. S. , Pirraglia, E. , Agüera‐Ortiz, L. F. , During, E. H. , Sacks, H. , Ayappa, I. , … de Leon, M. J. (2011). Greater risk of Alzheimer's disease in older adults with insomnia. Journal of the American Geriatrics Society, 59, 559–562. 10.1111/j.1532-5415.2010.03288.x 21391952 PMC3378676

[acel12802-bib-0123] O'Sullivan, R. J. , Kubicek, S. , Schreiber, S. L. , & Karlseder, J. (2010). Reduced histone biosynthesis and chromatin changes arising from a damage signal at telomeres. Nature Structural & Molecular Biology, 17, 1218–1225. 10.1038/nsmb.1897 PMC2951278 20890289

[acel12802-bib-0124] Ott, A. , Stolk, R. P. , van Harskamp, F. , Pols, H. A. , Hofman, A. , & Breteler, M. M. (1999). Diabetes mellitus and the risk of dementia: The Rotterdam study. Neurology, 53, 1937–1942. 10.1212/WNL.53.9.1937 10599761

[acel12802-bib-0125] Palop, J. J. , & Mucke, L. (2010). Amyloid‐β‐induced neuronal dysfunction in Alzheimer's disease: From synapses toward neural networks. Nature Neuroscience, 13, 812–818. 10.1038/nn.2583 20581818 PMC3072750

[acel12802-bib-0126] Paneni, F. , Diaz Cañestro, C. , Libby, P. , Lüscher, T. F. , & Camici, G. G. (2017). The aging cardiovascular system: Understanding it at the cellular and clinical levels. Journal of the American College of Cardiology, 69, 1952–1967. 10.1016/j.jacc.2017.01.064 28408026

[acel12802-bib-0127] Pawlikowska, L. , Hu, D. , Huntsman, S. , Sung, A. , Chu, C. , Chen, J. , … Study of Osteoporotic, F (2009). Association of common genetic variation in the insulin/IGF1 signaling pathway with human longevity. Aging Cell, 8, 460–472. 10.1111/j.1474-9726.2009.00493.x 19489743 PMC3652804

[acel12802-bib-0128] Peila, R. , Rodriguez, B. L. , & Launer, L. J. (2002). Type 2 diabetes, APOE gene, and the risk for dementia and related pathologies: The Honolulu‐Asia aging study. Diabetes, 51, 1256–1262. 10.2337/diabetes.51.4.1256 11916953

[acel12802-bib-0129] Peleg, S. , Sananbenesi, F. , Zovoilis, A. , Burkhardt, S. , Bahari‐Javan, S. , Agis‐Balboa, R. C. , … Fischer, A. (2010). Altered histone acetylation is associated with age‐dependent memory impairment in mice. Science (New York, NY), 328, 753–756. 10.1126/science.1186088 20448184

[acel12802-bib-0130] Perry, T. , Lahiri, D. K. , Sambamurti, K. , Chen, D. , Mattson, M. P. , Egan, J. M. , & Greig, N. H. (2003). Glucagon‐like peptide‐1 decreases endogenous amyloid‐beta peptide (Abeta) levels and protects hippocampal neurons from death induced by Abeta and iron. Journal of Neuroscience Research, 72, 603–612. 10.1002/jnr.10611 12749025

[acel12802-bib-0131] Petanceska, S. S. , & Gandy, S. (1999). The phosphatidylinositol 3‐kinase inhibitor wortmannin alters the metabolism of the Alzheimer's amyloid precursor protein. Journal of Neurochemistry, 73, 2316–2320. 10.1046/j.1471-4159.1999.0732316.x 10582589

[acel12802-bib-0132] Pistollato, F. , Sumalla Cano, S. , Elio, I. , Masias Vergara, M. , Giampieri, F. , & Battino, M. (2016). Role of gut microbiota and nutrients in amyloid formation and pathogenesis of Alzheimer disease. Nutrition Reviews, 74, 624–634. 10.1093/nutrit/nuw023 27634977

[acel12802-bib-0133] Prinz, P. N. , Vitaliano, P. P. , Vitiello, M. V. , Bokan, J. , Raskind, M. , Peskind, E. , & Gerber, C. (1982). Sleep, EEG and mental function changes in senile dementia of the Alzheimer's type. Neurobiology of Aging, 3, 361–370. 10.1016/0197-4580(82)90024-0 7170052

[acel12802-bib-0134] Prokop, S. , Miller, K. R. , & Heppner, F. L. (2013). Microglia actions in Alzheimer’s disease. Acta Neuropathologica, 126, 461–477. 10.1007/s00401-013-1182-x 24224195

[acel12802-bib-0135] Puglielli, L. , Konopka, G. , Pack‐Chung, E. , Ingano, L. A. , Berezovska, O. , Hyman, B. T. , … Kovacs, D. M. (2001). Acyl‐coenzyme A: Cholesterol acyltransferase modulates the generation of the amyloid beta‐peptide. Nature Cell Biology, 3, 905–912. 10.1038/ncb1001-905 11584272

[acel12802-bib-0136] Rasmussen, T. , Schliemann, T. , Sørensen, J. C. , Zimmer, J. , & West, M. J. (1996). Memory impaired aged rats: No loss of principal hippocampal and subicular neurons. Neurobiology of Aging, 17, 143–147. 10.1016/0197-4580(95)02032-2 8786797

[acel12802-bib-0137] Richetin, K. , Leclerc, C. , Toni, N. , Gallopin, T. , Pech, S. , Roybon, L. , & Rampon, C. (2015). Genetic manipulation of adult‐born hippocampal neurons rescues memory in a mouse model of Alzheimer's disease. Brain, 138, 440–455. 10.1093/brain/awu354 25518958

[acel12802-bib-0138] Richetin, K. , Petsophonsakul, P. , Roybon, L. , Guiard, B. P. , & Rampon, C. (2017). Differential alteration of hippocampal function and plasticity in females and males of the APPxPS1 mouse model of Alzheimer's disease. Neurobiology of Aging, 57, 220–231. 10.1016/j.neurobiolaging.2017.05.025 28666707

[acel12802-bib-0139] Riddell, D. R. , Christie, G. , Hussain, I. , & Dingwall, C. (2001). Compartmentalization of beta‐secretase (Asp2) into low‐buoyant density, noncaveolar lipid rafts. Current Biology, 11, 1288–1293. 11525745 10.1016/s0960-9822(01)00394-3

[acel12802-bib-0140] Rivera, E. J. , Goldin, A. , Fulmer, N. , Tavares, R. , Wands, J. R. , & de la Monte, S. M. (2005). Insulin and insulin‐like growth factor expression and function deteriorate with progression of Alzheimer's disease: Link to brain reductions in acetylcholine. Journal of Alzheimer's Disease, 8, 247–268. 10.3233/JAD-2005-8304 16340083

[acel12802-bib-0141] Roh, J. H. , Huang, Y. , Bero, A. W. , Kasten, T. , Stewart, F. R. , Bateman, R. J. , & Holtzman, D. M. (2012). Disruption of the sleep‐wake cycle and diurnal fluctuation of β‐amyloid in mice with Alzheimer’s disease pathology. Science Translational Medicine, 4, 150ra122‐150ra122. https://doi:10.1126/scitranslmed.3004291 10.1126/scitranslmed.3004291 PMC3654377 22956200

[acel12802-bib-0142] Salter, M. W. , & Stevens, B. (2017). Microglia emerge as central players in brain disease. Nature Medicine, 23, 1018–1027. 10.1038/nm.4397 28886007

[acel12802-bib-0143] Santoro, A. , Ostan, R. , Candela, M. , Biagi, E. , Brigidi, P. , Capri, M. , & Franceschi, C. (2017). Gut microbiota changes in the extreme decades of human life: A focus on centenarians. Cellular and Molecular Life Sciences, 75(1), 129–148. 10.1007/s00018-017-2674-y 29032502 PMC5752746

[acel12802-bib-0144] Scheff, S. W. , & Price, D. A. (2006). Alzheimer's disease‐related alterations in synaptic density: Neocortex and hippocampus. Journal of Alzheimer's Disease, 9, 101–115. 10.3233/JAD-2006-9S312 16914849

[acel12802-bib-0145] Sen, P. , Shah, P. P. , Nativio, R. , & Berger, S. L. (2016). Epigenetic mechanisms of longevity and aging. Cell, 166, 822–839. 10.1016/j.cell.2016.07.050 27518561 PMC5821249

[acel12802-bib-0146] Sepe, A. , Tchkonia, T. , Thomou, T. , Zamboni, M. , & Kirkland, J. L. (2011). Aging and regional differences in fat cell progenitors ‐ a mini‐review. Gerontology, 57, 66–75. 10.1159/000279755 20110661 PMC3031153

[acel12802-bib-0147] Seripa, D. , Franceschi, M. , Matera, M. G. , Panza, F. , Kehoe, P. G. , Gravina, C. , … Pilotto, A. (2006). Sex differences in the association of apolipoprotein E and angiotensin‐converting enzyme gene polymorphisms with healthy aging and longevity: A population‐based study from Southern Italy. Journals of Gerontology. Series A, Biological Sciences and Medical Sciences, 61, 918–923. 10.1093/gerona/61.9.918 16960022

[acel12802-bib-0148] Sexton, C. E. , Betts, J. F. , Demnitz, N. , Dawes, H. , Ebmeier, K. P. , & Johansen‐Berg, H. (2016). A systematic review of MRI studies examining the relationship between physical fitness and activity and the white matter of the ageing brain. NeuroImage, 131, 81–90. 10.1016/j.neuroimage.2015.09.071 26477656 PMC4851455

[acel12802-bib-0149] Shanley, L. J. , Irving, A. J. , & Harvey, J. (2001). Leptin enhances NMDA receptor function and modulates hippocampal synaptic plasticity. Journal of Neuroscience, 21, Rc186 10.1523/JNEUROSCI.21-24-j0001.2001 11734601 PMC6763052

[acel12802-bib-0150] Shaw, A. C. , Goldstein, D. R. , & Montgomery, R. R. (2013). Age‐dependent dysregulation of innate immunity. Nature Reviews Immunology, 13(12), 875–887. 10.1038/nri3547 PMC4096436 24157572

[acel12802-bib-0151] Shinohara, M. , Kanekiyo, T. , Yang, L. , Linthicum, D. , Shinohara, M. , Fu, Y. , … Bu, G. (2016). APOE2 eases cognitive decline during aging: Clinical and preclinical evaluations. Annals of Neurology, 79, 758‐774. 10.1002/ana.24628 26933942 PMC5010530

[acel12802-bib-0152] Simons, M. , Keller, P. , De Strooper, B. , Beyreuther, K. , Dotti, C. G. , & Simons, K. (1998). Cholesterol depletion inhibits the generation of beta‐amyloid in hippocampal neurons. Proceedings of the National Academy of Sciences of the United States of America, 95, 6460–6464. 9600988 10.1073/pnas.95.11.6460 PMC27798

[acel12802-bib-0153] Sorrells, S. F. , Paredes, M. F. , Cebrian‐Silla, A. , Sandoval, K. , Qi, D. , Kelley, K. W. , … Alvarez‐Buylla, A. (2018). Human hippocampal neurogenesis drops sharply in children to undetectable levels in adults. Nature, 555, 377–381. 10.1038/nature25975 29513649 PMC6179355

[acel12802-bib-0154] St‐Amour, I. , Cicchetti, F. , & Calon, F. (2016). Immunotherapies in Alzheimer's disease: Too much, too little, too late or off‐target? Acta Neuropathologica, 131, 481–504. 10.1007/s00401-015-1518-9 26689922

[acel12802-bib-0155] Steen, E. , Terry, B. M. , Rivera, E. J. , Cannon, J. L. , Neely, T. R. , Tavares, R. , … de la Monte, S. M. (2005). Impaired insulin and insulin‐like growth factor expression and signaling mechanisms in Alzheimer's disease–is this type 3 diabetes? Journal of Alzheimer's Disease, 7, 63–80. 10.3233/JAD-2005-7107 15750215

[acel12802-bib-0156] Streit, W. J. , Sammons, N. W. , Kuhns, A. J. , & Sparks, D. L. (2004). Dystrophic microglia in the aging human brain. Glia, 45, 208–212. 10.1002/glia.10319 14730714

[acel12802-bib-0157] Strittmatter, W. J. , Weisgraber, K. H. , Huang, D. Y. , Dong, L. M. , Salvesen, G. S. , Pericak‐Vance, M. , … Roses, A. D. (1993). Binding of human apolipoprotein E to synthetic amyloid beta peptide: Isoform‐specific effects and implications for late‐onset Alzheimer disease. Proceedings of the National Academy of Sciences, 90, 8098–8102. 10.1073/pnas.90.17.8098 PMC47295 8367470

[acel12802-bib-0158] Tabuchi, M. , Lone, S. R. , Liu, S. , Liu, Q. , Zhang, J. , Spira, A. P. , & Wu, M. N. (2015). Sleep interacts with Aβ to modulate intrinsic neuronal excitability. Current Biology, 25, 702–712. 10.1016/j.cub.2015.01.016 25754641 PMC4366315

[acel12802-bib-0159] Taddei, S. , Virdis, A. , Ghiadoni, L. , Salvetti, G. , Bernini, G. , Magagna, A. , & Salvetti, A. (2001). Age‐related reduction of NO availability and oxidative stress in humans. Hypertension, 38, 274–279. 10.1161/01.HYP.38.2.274 11509489

[acel12802-bib-0160] Talbot, K. , Wang, H. Y. , Kazi, H. , Han, L. Y. , Bakshi, K. P. , Stucky, A. , … Arnold, S. E. (2012). Demonstrated brain insulin resistance in Alzheimer's disease patients is associated with IGF‐1 resistance, IRS‐1 dysregulation, and cognitive decline. Journal of Clinical Investigation, 122, 1316–1338. 10.1172/JCI59903 22476197 PMC3314463

[acel12802-bib-0161] Tan, L. , Yu, J.‐T. , Hu, N. , & Tan, L. (2013). Non‐coding RNAs in Alzheimer's disease. Molecular Neurobiology, 47, 382–393. 10.1007/s12035-012-8359-5 23054683

[acel12802-bib-0162] Thevaranjan, N. , Puchta, A. , Schulz, C. , Naidoo, A. , Szamosi, J. C. , Verschoor, C. P. , … Bowdish, D. M. E. (2017). Age‐associated microbial dysbiosis promotes intestinal permeability, systemic inflammation, and macrophage dysfunction. Cell Host & Microbe, 21, 455–466.e454. 10.1016/j.chom.2017.03.002 28407483 PMC5392495

[acel12802-bib-0163] Tolppanen, A. M. , Solomon, A. , Kulmala, J. , Kareholt, I. , Ngandu, T. , Rusanen, M. , … Kivipelto, M. (2015). Leisure‐time physical activity from mid‐ to late life, body mass index, and risk of dementia. Alzheimer's & Dementia : the Journal of the Alzheimer's Association, 11, 434–443.e436. 10.1016/j.jalz.2014.01.008 24721528

[acel12802-bib-0164] Tremlett, H. , Bauer, K. C. , Appel‐Cresswell, S. , Finlay, B. B. , & Waubant, E. (2017). The gut microbiome in human neurological disease: A review: Gut microbiome. Annals of Neurology, 81(3), 369–382. 10.1002/ana.24901 28220542

[acel12802-bib-0165] Tschudi, M. R. , Barton, M. , Bersinger, N. A. , Moreau, P. , Cosentino, F. , Noll, G. , … Lüscher, T. F. (1996). Effect of age on kinetics of nitric oxide release in rat aorta and pulmonary artery. Journal of Clinical Investigation, 98(4), 899–905. 10.1172/JCI118872 8770860 PMC507503

[acel12802-bib-0166] Vemuri, P. , Knopman, D. S. , Lesnick, T. G. , Przybelski, S. A. , Mielke, M. M. , Graff‐Radford, J. , … Jack, C. R. (2017). Evaluation of amyloid protective factors and Alzheimer Disease neurodegeneration protective factors in elderly individuals. JAMA Neurology, 74(6), 718–726. 10.1001/jamaneurol.2017.0244 28418521 PMC5649401

[acel12802-bib-0167] Vemuri, P. , Lesnick, T. G. , Przybelski, S. A. , Knopman, D. S. , Lowe, V. J. , Graff‐Radford, J. , … Jack, C. R. (2017). Age, vascular health, and Alzheimer's disease biomarkers in an elderly sample. Annals of Neurology, 82, 706‐718. 10.1002/ana.25071 29023983 PMC5696029

[acel12802-bib-0168] Vetrivel, K. S. , & Thinakaran, G. (2010). Membrane rafts in Alzheimer’s disease beta‐amyloid production. Biochimica Et Biophysica Acta, 1801, 860–867. 10.1016/j.bbalip.2010.03.007 20303415 PMC2886169

[acel12802-bib-0169] Vidoni, E. D. , Van Sciver, A. , Johnson, D. K. , He, J. , Honea, R. , Haines, B. , … Burns, J. M. (2012). A community‐based approach to trials of aerobic exercise in aging and Alzheimer's disease. Contemporary Clinical Trials, 33, 1105–1116. 10.1016/j.cct.2012.08.002 22903151 PMC3468654

[acel12802-bib-0170] Villafuerte, G. , Miguel‐Puga, A. , Murillo Rodríguez, E. , Machado, S. , Manjarrez, E. , & Arias‐Carrión, O. (2015). Sleep deprivation and oxidative stress in animal models: A systematic review. Oxidative Medicine and Cellular Longevity, 2015, 1–15. 10.1155/2015/234952 PMC4402503 25945148

[acel12802-bib-0171] Vita, J. A. , Treasure, C. B. , Nabel, E. G. , McLenachan, J. M. , Fish, R. D. , Yeung, A. C. , … Ganz, P. (1990). Coronary vasomotor response to acetylcholine relates to risk factors for coronary artery disease. Circulation, 81, 491–497. 10.1161/01.CIR.81.2.491 2105174

[acel12802-bib-0172] Wahrle, S. , Das, P. , Nyborg, A. C. , McLendon, C. , Shoji, M. , Kawarabayashi, T. , … Golde, T. E. (2002). Cholesterol‐dependent gamma‐secretase activity in buoyant cholesterol‐rich membrane microdomains. Neurobiology of Diseases, 9, 11–23. 10.1006/nbdi.2001.0470 11848681

[acel12802-bib-0173] Whitmer, R. A. , Gustafson, D. R. , Barrett‐Connor, E. , Haan, M. N. , Gunderson, E. P. , & Yaffe, K. (2008). Central obesity and increased risk of dementia more than three decades later. Neurology, 71, 1057–1064. 10.1212/01.wnl.0000306313.89165.ef 18367704

[acel12802-bib-0174] Willcox, B. J. , Donlon, T. A. , He, Q. , Chen, R. , Grove, J. S. , Yano, K. , … Curb, J. D. (2008). FOXO3A genotype is strongly associated with human longevity. Proceedings of the National Academy of Sciences of the United States of America, 105, 13987–13992. 10.1073/pnas.0801030105 18765803 PMC2544566

[acel12802-bib-0175] Wirth, M. , Haase, C. M. , Villeneuve, S. , Vogel, J. , & Jagust, W. J. (2014). Neuroprotective pathways: Lifestyle activity, brain pathology, and cognition in cognitively normal older adults. Neurobiology of Aging, 35, 1873–1882. 10.1016/j.neurobiolaging.2014.02.015 24656834 PMC4019766

[acel12802-bib-0176] Wyss‐Coray, T. , & Rogers, J. (2012). Inflammation in Alzheimer disease‐a brief review of the basic science and clinical literature. Cold Spring Harb Perspect Med, 2, a006346 10.1101/cshperspect.a006346 22315714 PMC3253025

[acel12802-bib-0177] Xie, L. , Kang, H. , Xu, Q. , Chen, M. J. , Liao, Y. , Thiyagarajan, M. , … Nedergaard, M. (2013). Sleep drives metabolite clearance from the adult brain. Science, 342, 373–377. 10.1126/science.1241224 24136970 PMC3880190

[acel12802-bib-0178] Yaffe, K. , Laffan, A. M. , Harrison, S. L. , Redline, S. , Spira, A. P. , Ensrud, K. E. , … Stone, K. L. (2011). Sleep disordered breathing, hypoxia, and risk of mild cognitive impairment and dementia in older women. Jamathe Journal of the American Medical Association, 306, 613–619. 10.1001/jama.2011.1115 21828324 PMC3600944

[acel12802-bib-0179] Yamada, T. , Akiyama, H. , & McGeer, P. L. (1990). Complement‐activated oligodendroglia: A new pathogenic entity identified by immunostaining with antibodies to human complement proteins C3d and C4d. Neuroscience Letters, 112, 161–166. 10.1016/0304-3940(90)90196-G 2359515

[acel12802-bib-0180] Yu, L. , Chibnik, L. B. , Yang, J. , McCabe, C. , Xu, J. , Schneider, J. A. , … Bennett, D. A. (2016). Methylation profiles in peripheral blood CD4+ lymphocytes versus brain: The relation to Alzheimer's disease pathology. Alzheimer's & Dementia: the Journal of the Alzheimer's Association, 12, 942–951. 10.1016/j.jalz.2016.02.009 PMC5014706 27016692

[acel12802-bib-0181] Zhang, B. , Gaiteri, C. , Bodea, L.‐G. , Wang, Z. , McElwee, J. , Podtelezhnikov, A. A. , … Emilsson, V. (2013). Integrated systems approach identifies genetic nodes and networks in late‐onset Alzheimer's disease. Cell, 153, 707–720. 10.1016/j.cell.2013.03.030 23622250 PMC3677161

[acel12802-bib-0182] Zhang, Y. , Kim, M. S. , Jia, B. , Yan, J. , Zuniga‐Hertz, J. P. , Han, C. , & Cai, D. (2017). Hypothalamic stem cells control ageing speed partly through exosomal miRNAs. Nature, 548, 52–57. 10.1038/nature23282 28746310 PMC5999038

[acel12802-bib-0183] Zhang, K. , Schrag, M. , Crofton, A. , Trivedi, R. , Vinters, H. , & Kirsch, W. (2012). Targeted proteomics for quantification of histone acetylation in Alzheimer's disease. Proteomics, 12, 1261–1268. 10.1002/pmic.201200010 22577027 PMC6812507

[acel12802-bib-0184] Zhao, H.‐Q. , Zhang, P. , Gao, H. , He, X. , Dou, Y. , Huang, A. Y. , … Wei, L. (2015). Profiling the RNA editomes of wild‐type *C. elegans* and ADAR mutants. Genome Research, 25, 66–75. 10.1101/gr.176107.114 25373143 PMC4317174

[acel12802-bib-0185] Zhao, J. , Zhu, Y. , Yang, J. , Li, L. , Wu, H. , De Jager, P. L. , … Bennett, D. A. (2017). A genome‐wide profiling of brain DNA hydroxymethylation in Alzheimer's disease. Alzheimer's & Dementia: the Journal of the Alzheimer's Association, 13, 674–688. 10.1016/j.jalz.2016.10.004 PMC6675576 28089213

